# An immunoregulatory and metabolism-improving injectable hydrogel for cardiac repair after myocardial infarction

**DOI:** 10.1093/rb/rbae131

**Published:** 2024-11-13

**Authors:** Yage Sun, Xinrui Zhao, Qian Zhang, Rong Yang, Wenguang Liu

**Affiliations:** School of Materials Science and Engineering, Tianjin Key Laboratory of Composite and Functional Materials, Tianjin University, Tianjin 300350, China; School of Materials Science and Engineering, Tianjin Key Laboratory of Composite and Functional Materials, Tianjin University, Tianjin 300350, China; School of Materials Science and Engineering, Tianjin Key Laboratory of Composite and Functional Materials, Tianjin University, Tianjin 300350, China; School of Materials Science and Engineering, Tianjin Key Laboratory of Composite and Functional Materials, Tianjin University, Tianjin 300350, China; School of Materials Science and Engineering, Tianjin Key Laboratory of Composite and Functional Materials, Tianjin University, Tianjin 300350, China

**Keywords:** injectable hydrogel, myocardial infarction, immunoregulatory, glucose metabolism, cardiac fibrosis

## Abstract

The hypoxia microenvironment post-myocardial infarction (MI) critically disturbs cellular metabolism and inflammation response, leading to scarce bioenergy supplying, prolonged inflammatory phase and high risk of cardiac fibrosis during cardiac restoration. Herein, an injectable hydrogel is prepared by Schiff base reaction between fructose-1,6-bisphosphate (FBP)-grafted carboxymethyl chitosan (CF) and oxidized dextran (OD), followed by loading fucoidan-coated baicalin (BA)-encapsulated zein nanoparticles (BFZ NPs), in which immunoregulatory and metabolism improving functions are integrally included. The grafted FBP serves to enhance glycolysis and provide more bioenergy for cardiomyocytes survival under hypoxia microenvironment, and elevating cellular antioxidant capacity *via* pentose phosphate pathway. OD with intrinsic anti-inflammatory effect can induce M2 polarization of macrophages to accelerate inflammatory elimination. While facing the possibility of endothelial-to-mesenchymal transition (EndoMT) caused by excessive expressed TGF-β1 secreted from M2 macrophages, BFZ NPs can target endothelia cells and intracellularly release BA to regulate the level of fatty acid oxidation, resulting in retained endothelial features and decreased risk of cardiac fibrosis. After being injecting the hydrogel into rats’ infarcted cardiac, the 28-day-post surgical outcomes demonstrate its benign effects on restoring cardiac functions and attenuating adverse left ventricular remodeling. This study shows a promising measure for MI treatment with immunoregulating and metabolism regulation comprehensively.

## Graphical abstract

**Figure:**
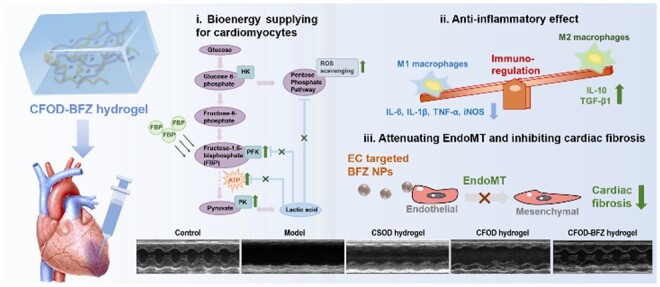

## Introduction

Myocardial infarction (MI) has long been regarded as a high-risk cardiovascular disease due to its high morbidity and mortality, which severely poses a threat to human health [1–3]. In consideration of the pathological repairing process of MI that is divided into inflammatory phase, proliferative phase and remodeling phase [4], a regular and balanced inflammatory response is essential to initiate the following reparative process, making inflammatory regulation become the primary goal in myocardial tissue engineering [5]. Several previous studies have confirmed the treating efficiency of small molecular antioxidant or anti-inflammatory drugs loaded by biomaterials [6–8], but they still face dilemma in achieving long period therapies due to their short half-life time and retention time at the infarcted sites. Thereupon, self-immunoregulating hydrogels originated from anti-inflammatory polymers have drawn great attention currently, wherein various biocompatible polysaccharides, such as fucoidan, laminarin and dextran, have been researched in recent work, presenting effective and long-term functions on inducing M2 phenotype of macrophages and promoting the release of plentiful anti-inflammatory cytokines [9–11].

However, the abundantly secretive cytokines from M2 macrophages do not always show positive effects on cardiac reparation. Recent studies have reported that transforming growth factor-β1 (TGF-β1) can give rise to endothelial-to-mesenchymal transition (EndoMT) [12], which means that the endotheliocytes loss their endothelial features and some characteristics of mesenchymal cells or fibroblasts emerge, further leading to the occurrence of atherosclerosis or cardiac fibrosis to aggravate the adverse left ventricular remodeling post-MI [13, 14]. Previous studies have verified that the pathological change of EndoMT can be attributed to the blockage of carnitine palmitoyltransferase 1A (CPT1A) under the role of TGF-β1, which is a rate-limiting enzyme in fatty acid oxidation (FAO). And the inhibited FAO further can regulate the intracellular acetyl-coenzyme A level and SMAD7 signaling to bring about EndoMT [15]. Aiming at the underlying targets along this pathway, several strategies have been proposed to prohibit the unexpected EndoMT, including increasing CPT1A level by apolipoprotein E or baicalin treating [16, 17], exogenously supplementing citrate to enhance the activity of acetyl-CoA [18, 19] and inhibiting the intracellular accumulation of fatty acid [14], ultimately to avoid the undesirable cardiac fibrosis and cardiac dysfunction caused by excessively expressed TGF-β1 originated from M2 macrophages. However, most of the studies still stay on developing the molecular mechanism of the drugs, and few of them have been used in concrete therapy for tissue repair *via* hydrogel mediated drug delivery.

Meanwhile, cardiomyocytes experience ischemia and hypoxia to a great extent during MI, impacting their glucose metabolism to be altered toward glycolysis for cellular energy supplying, which produces much less adenosine triphosphate (ATP) compared with aerobic pathway [20–22]. Moreover, accumulated lactic acid produced from glycolysis will decrease the activity of phosphofructokinase (PFK) and pyruvate kinase (PK) *via* negative feedback mechanism to further reduce the production of ATP, leading to expedited apoptosis of cardiomyocytes and impeded cardiac repairing process [23–25]. In order to restore ATP providing to a normal level, a high-energy intermediate of glycolysis—fructose-1,6-bisphosphate (FBP) has been considered to be additionally supplemented to cardiomyocytes, which is expected to enhance the activity of PFK and PK and maintain the regular cycle of glycolysis, showing advantages in rescuing the cardiomyocytes from hypoxic and ischemic conditions. However, the traditionally intravenous injection of FBP usually inquires a relatively high dose due to its non-targeted administration and thus elicits the risk of lactic acid poisoning [26]. Therefore, locally delivering FBP to the injured tissues through *in situ* injection arises to be a potential therapeutic strategy. A current method for *in situ* injection of FBP is to introduce Cu ions for inducing gelation via and coordinative bond PO_4_^3−^–Cu^2+^, while the existence of Cu ions still faces the risk of biosafety [27].

**Figure 1.**
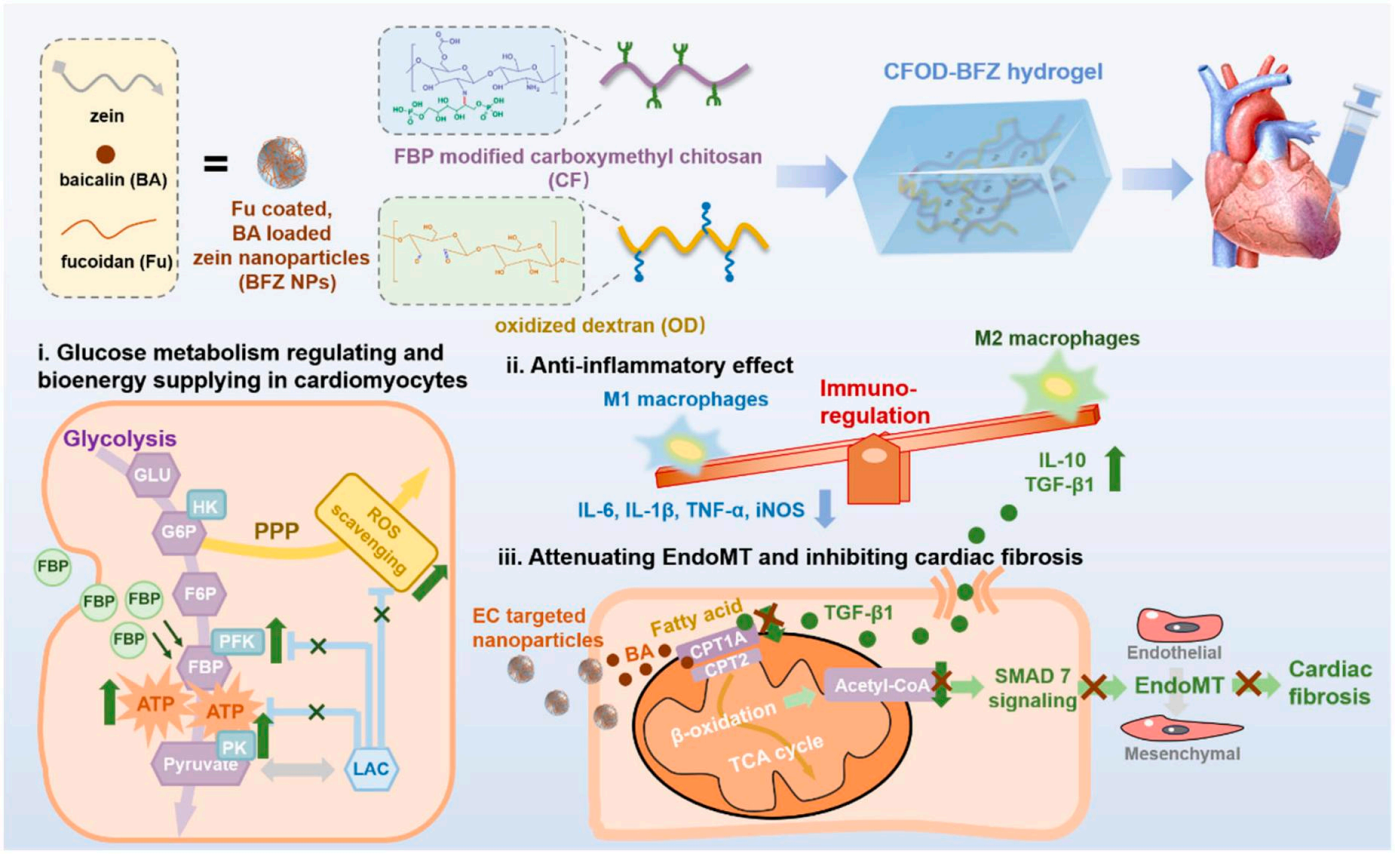
Schematic illustration of preparation of the injectable hydrogel with *in situ* cellular energy supplying and self-immunoregulating functions to enhance the viability of cardiomyocytes, eliminate excessive inflammatory response and attenuate cardiac fibrosis led by EndoMT.

Taking all the discussions above into account, we designed and prepared an *in situ* cellular energy supplying and self-immunoregulating injectable nanocomposite hydrogel in this work, aiming to provide sufficient bioenergy for cardiomyocytes suffering high oxidative stress post-MI, and regulate the inflammatory response without inducing cardiac fibrosis caused by EndoMT (Figure 1). To achieve the self-immunoregulating goal, the dextran with a molecular weight of 40 kDa was chosen for its satisfying antioxidant and anti-inflammatory properties [28, 29] and was partially oxidized to form the aldehyde groups (OD) for further crosslinking. As for *in situ* providing FBP for cellular glycolysis, FBP was grafted onto the side chains of carboxymethyl chitosan (CS), forming a Schiff base derivative (CF) [30, 31]. Additionally, baicalin (BA) was a major flavonoid component of the traditional Chinese medicine *Scutellaria baicalensis*, which showed a strong effect of lipid-reducing. In a recent study, the underlying mechanism of its lipid-reducing effect had been verified. The baicalin could directly activate CPT1A as its target to accelerate the influx rate of long-chain acyl-CoAs into mitochondria for β-oxidation [17]. Thus, the introduction of BA into this hydrogel system was expected to improve the CPT1A level for fatty acid oxidation and prohibit the potential undesired EndoMT process, which would ultimately show effects in attenuating the adverse left ventricular remodeling; nonetheless the solubility of BA in water and sustained releasing behavior still needed to be improved as a hydrophobic small molecular drug. Biocompatible zein was considered to be an appropriate carrier for loading BA, since it could self-assemble into nanoparticles in water [32, 33]. However, the weak acid microenvironment post-MI was just around the isoelectric point of zein, making the self-assembled zein nanoparticles unstable and tend to aggregate, which could be effectively overcome through complexing anionic polysaccharides [34, 35]. The p*K*a value of sulfate groups in fucoidan (Fu) is around 2. When the pH value was higher than the p*K*a of Fu but lower than the isoelectric point of zein (which is between 2 and 6), Fu was ionized with negative charges and has the potential to stabilize zein nanoparticles by forming a negatively charged outer layer to enhance the repulsion among each particle. Thus, Fu with L-fucopyranose units and sulfated ester groups was an ideal choice for stabling zein nanoparticles *via* electrostatic interaction and hydrogen bonds [36, 37]. More significantly, Fu possessed endothelial-targeting character since it could competitively bind with the P-selectin expressed on the surface of human umbilical vein endothelia cells (HUVECs), especially in the inflammatory microenvironment [38, 39]. Ultimately, the Fu-coated and BA-loaded zein self-assembled nanoparticles (BFZ NPs) would show advantages in elevating endothelial CPT1A activity to facilitate FAO and block unexpected EndoMT stimulated by overly expressed TGF-β1 during cardiac reparation through targeting HUVECs and intracellular delivering BA. The final formed nanocomposite hydrogel (CFOD-BFZ hydrogel) possessed rapid gelation time through Schiff base reaction between CF and OD. It was expected to provide injured cardiomyocytes with more bioenergy *via in situ* glycolysis improving, regulate the inflammatory and oxidative microenvironment and inhibit cardiac fibrosis arising from EndoMT caused by abundant TGF-β1 cytokines, which would promote the cardiac functions and attenuate the adverse left ventricular remodeling synergistically in an acute MI model.

## Materials and methods

### Materials

Dextran (MW = 40 kDa), sodium periodate (NaIO_4_), carboxymethyl chitosan (CS) and D-fructose-1,6-diphosphate trisodium salt octahydrate were purchased from Macklin (Shanghai, China). Fucoidan (Mw = 276 kDa, sulfate: 29.65%) powder was purchased from Bright Moon Seaweed Group Co., Ltd (Qingdao, China). Zein, coumarin and baicalin (BA) were purchased from Heowns Biochem Technologies Co., Ltd (Tianjin, China). Safranine O (85%), ferrous sulfate heptahydrate (FeSO_4_·7H_2_O, 99%), hydrogen peroxide (H_2_O_2_, 30 wt%), 1,1-diphenyl-2-picrylhydrazyl free radical (DPPH, 97%) were purchased from Aladdin (Shanghai, China). 4,6-Diamidino-2-phenylindole (DAPI) and 3-(4,5-dimethyl-2-thiazolyl)-2,5-diphenyl-2-H- tetrazolium bromide (98%, MTT), TRIzol reagent, Hoechst 33342, lipopolysaccharide (LPS, 99%) and TGF-β1 ELISA assay kit were purchased from Solarbio life Sciences (Beijing, China). The ATP content assay kit, PFK activity assay kit, PK activity assay kit, G6PDH activity assay kit, 2′,7′-dichlorodihydrofluorescein diacetate (DCFH-DA) and total antioxidant capacity assay kit were purchased from Beyotime (Wuhan, China). Anti-CD31, anti-CD44, anti-TGF-β1 and m-lgG Fc BP-HRP were purchased from MedChemExpress LLC (Shanghai, China).

### Cell culture

H9C2 cells and Raw 264.7 cells were both purchased from EK-Bioscience (Shanghai, China) and cultured in DMEM medium with 10% FBS and 1% P/S. HUVECs (EK-Bioscience, Shanghai, China) were cultured in ECM medium with 5% FBS, 1% P/S and 1% ECGS additively. All of the cells were cultured in a 37 °C incubator contained 5% CO_2_ and suitable humidity.

### Preparation and characterization of BZ NPs and BFZ NPs

The BA-zein nanoparticles (BZ NPs) and BFZ NPs were obtained mainly *via* the self-assemble capacity of zein in water. Briefly, 100 mg zein and 4 mg (2 mM) BA were dissolved in 10 ml 80% ethanol solution to form a uniform liquor. Meanwhile, 50 ml pure water or Fu solution (different mass ratio with zein) was prepared, and the pH was adjusted to 3.5 with citric acid. Subsequently, the zein/BA ethanol solution was dropwise added into the pure water or Fu solution prepared above. Finally, the ethanol was removed through rotary evaporation to form BZ NPs for control and BFZ NPs. The particle size and zeta potential of the nanoparticles were detected by dynamic light scattering (DLS) instrument and Zetasizer Nano ZS instrument (Nano-ZS90, Malvern, England). TEM (Jem-2100f, JEOL, Japan) was used to observe the morphology.

For cellular uptake tests, the coumarin (Co6) was loaded in the nanoparticles to provide the fluorescence for observation due to the intrinsic fluorescence property. In brief, H9C2 cells, raw 264.7 cells and HUVECs were, respectively, cultured in a 24-well plate at the density of 1 × 10^5^ for 24 h, followed by adding 50 μl Co6 loaded BZ NPs or BFZ NPs solution and incubating for another 2 h. Before observation, the nucleus was stained with Hoechst 33342 and the cells were photographed with an inverted fluorescence microscope (Invitrogen, EVOS M5000, USA).

The loading capacity and encapsulation efficiency of the nanoparticles were measured by Ultra-Violet Spectrometer (Genesys 180, Thermo Scientific) at 246 nm and calculated by the following equation:

(1) $$\mathrm{Loading}\;\mathrm{capacity}\;\%=\;\frac{\mathrm{mass}\;\mathrm{of}\;\mathrm{loaded}\;\mathrm{BA}}{\mathrm{total}\;\mathrm{mass}\;\mathrm{of}\;\mathrm{BFZ}\;\mathrm{NPs}}\;\times \;100\%$$

 

(2) $$\mathrm{Encapsulation}\;\mathrm{efficiency}\;\%=\;\frac{\mathrm{mass}\;\mathrm{of}\;\mathrm{loaded}\;\mathrm{BA}}{\mathrm{total}\;\mathrm{mass}\;\mathrm{of}\;\mathrm{added}\;\mathrm{BA}}\;\times \;100\%$$

### Preparation and characterization of OD

OD was synthesized referring to the previously study [3]. Briefly, dextran (1.50 g, 6.19 mmol) was dissolved in 150 ml deionized water followed by adding 1.59 g NaIO_4_ (15.47 mmol) to the above aqueous solution. The reaction mixture was stirred for 4 h at room temperature and stopped by dropping 30 ml ethylene glycol. The solution was then dialyzed against DI water with a dialysis tube (3.5 kDa) for 3 days and then lyophilized for further use. The structure of OD was characterized by Fourier transform infrared spectrometry (FTIR, Nicolet 6700, Thermo Scientific).

### Preparation and characterization of CF

The synthesis of CF relied on the Schiff base reaction between the carbanyl groups in FBP and the amino groups in CS according to the previous works [30, 31]. First, CS (1.00 g, 1.8 mmol) was dissolved in 100 ml DI water, followed by adding 3.04 g (5.4 mmol) FBP under stirring. The reaction was continued for 72 h at 37 °C in dark. Finally, it was dialyzed against DI water with a dialysis tube (3.5 kDa) for 3 days followed by lyophilization for use. The structure of CF was characterized by ^1^H NMR (AVANCE III, 400 MHz, Bruker) using D_2_O as a solvent, Fourier transform infrared spectrometry (FTIR, Nicolet 6700, Thermo Scientific) and X-ray diffractometer (XRD, D8 advanced, Brucker).

### Preparation and characterizations of the injectable hydrogels

The CFOD hydrogel was formed through the Schiff base reaction between the aldehyde groups on OD and the amino acid groups on CF by mixing 5 wt% OD solution and 10 wt% CF solution with equal volume. As for CFOD-BFZ hydrogel, the OD was pre-mixed with 2.5 mg/ml BFZ NPs, and then mixed with CF solution for preparing CFOD-BFZ hydrogel. In the part of cellular experiments and *in vivo* experiments, the CSOD hydrogel and CSOD-BFZ hydrogel were applied as control, which were prepared by substituting the CF component with CS at the same concentration.

The gelation time of the hydrogels was observed by vial inversion method. The rheological properties were measured by Anton Paar MCR302 (Austria) with a 25 mm diameter parallel plate and a gap of 0.8 mm at 37 °C. The time sweeping was conducted over a time range from 0 to 180 s with 1% strain and the frequency of 1 Hz. The frequency sweeping was carried out from 0.1 to 10 Hz. To verify the shear-thinning properties of the hydrogels, a shear-thinning test was conducted with the shear rate changing from 0.1 to 100 s^−1^. The self-recovery property was verified by varying the shear strain from 1% to 100–300% with a time interval of 60 s, in which the storage modulus G′ and the loss modulus G″ were recorded.

The reactive oxide species (ROS) scavenging ability of CFOD-BFZ hydrogel was evaluated by measuring their inhibition effect on DPPH radicals, hydroxyl radicals and hydrogen peroxide radicals. In detail, 5 ml DPPH methanol solution and 200 μl 5 wt% OD solution or 200 mg hydrogel were mixed, respectively. The supernatant was taken out at the specific time and the absorbance was measured at the wavelength of 520 nm. The control group was prepared by adding 200 μl 80% methanol into the DPPH solution. The scavenging ability was calculated using the following equation:

(3) $$\mathrm{Scavenging}\;\mathrm{ability}=(1\;-\;\frac{A\;\text{sample}}{A\;\text{contro}l})\;\times \;100\%$$

where A sample and A control are the absorbance of the sample and control.

The scavenging effect on hydroxyl radicals was evaluated by Fenton reaction. Briefly, 600 μl FeSO_4_ solution (2 mmol/l), 500 μl safranin O solution (360 μg/ml) and 300 μl samples (OD solution or CFOD-BFZ hydrogel or DI water as blank group) were mixed and incubated for 10 min. Subsequently, 800 μl 6 wt% H_2_O_2_ solution was added and incubated for another 30 min at 55 °C. In the control group, 800 μl deionized water was added to substitute the H_2_O_2_ solution. The absorbance of the supernatant was examined at 492 nm on a microplate reader (Infinite M200 PRO, Tecan, Switzerland). The scavenging ability was calculated using the following equation:

(4) $$\mathrm{Scavenging}\;\mathrm{ability}=\frac{A\;\text{sample}-A\;\text{blank}\;}{A\;\text{contro}l-A\;\text{blank}}\;\times \;100\%$$

where A sample, A control and A blank are the absorbance values of hydrogel, control and blank groups.

The scavenging effect on hydrogen peroxide radicals was investigated by first mixing 400 μl samples (OD solution or CFOD-BFZ hydrogel or DI water as control group) with equal volume 0.2 M H_2_O_2_ solution, followed by adding the solution above into 2.6 ml 1 mg/mL TiSO_4_ solution. After incubating the mixture for 60 min at 37 °C, the absorbance of the supernatant was examined at 405 nm on a microplate reader (Infinite M200 PRO, Tecan, Switzerland). The scavenging ability was calculated using the following equation:

(5) $$\mathrm{Scavenging}\;\mathrm{ability}=(1\;-\;\frac{A\;\text{sample}}{A\;\text{contro}l})\;\times \;100\%$$

where A sample and A control are the absorbance values of the hydrogel and control groups.

### Investigation of the BA releasing behavior from CFOD-BFZ hydrogel

About 400 μL CFOD-BFZ hydrogels were immersed into 8 ml PBS or MES (pH = 6.0) and incubated at 37 °C to mimic the normal physiological microenvironment or oxidative microenvironment, respectively. At the specific time points, 2 ml supernatant was collected and another 2 ml fresh PBS or MES solution was added. The collected supernatant was then lyophilized and redissolved with ethanol. The absorption at 246 nm was detected by Ultra-Violet Spectrometer (Genesys 180, Thermo Scientific), and the BA releasing behavior was then calculated.

### Biocompatibility assay of the CFOD and CFOD-BFZ hydrogel

The cytotoxicity of the hydrogels was evaluated using MTT assay with H9C2 cells. The sterile hydrogels were immersed in the culture medium at the concentration of 0.1 g/ml for 24 h to obtain the leaching solution. The H9C2 cells were seeded in 96-well plates at the density of 2 × 10^4^ cells per well and incubated for 24 h. Then, the culture medium was removed and refreshed with 200 μl leaching solution. After culturing for 24, 72 and 120 h, the medium was replaced with the mixture of culture medium and MTT (*V*:*V* = 9:1), and incubated for another 4 h. Finally, the medium was removed and 200 μl DMSO was added into the plate. The absorbance was measured at 570 nm by Model 550 microplate reader (Bio-Rad, USA). The non-treated cells were set as control and the cell viability was calculated as following:

(6) $$\mathrm{Cell}\;\mathrm{viability}=\frac{A\;\text{sample}}{A\;\text{contro}l}\;\times \;100\%$$

where A sample and A control represents the absorbances of the experimental group and the control group, respectively.

Hemolysis test was performed according to the following steps. About 2 ml PBS, 20 μl erythrocytes and 200 μl CFOD hydrogel or CFOD-BFZ hydrogel were mixed as experimental groups. About 20 μl erythrocytes were added into 2 ml deionized water to serve as a positive control group, and 2 ml of saline with 20 μl erythrocytes was set as negative control. After incubating at 37 °C for 2 h, the mixed solutions were centrifuged at 2000 rpm for 10 min. The absorbance of the supernatant was tested at 545 nm by Model 550 microplate reader (Bio-Rad, USA). And the hemolysis ratio was calculated as the following equation:

(7) $$\mathrm{Hemolysis}\;\mathrm{ratio}=\;\frac{A\;s\text{ample}-A\;\text{nc}\;}{A\;\text{pc}-A\;\text{nc}}\;\times \;100\%$$

where A sample, A nc and A pc, respectively, represent the absorbance at 545 nm of the sample groups, the negative control group and the positive control group.

### *In vitro* migration assay of H9C2 cells

The scratch wound healing test was conducted to assay the migration capacity of H9C2 cells under oxidative condition with the treatment of the CFOD-BFZ hydrogel. About 1 × 10^5^ H9C2 cells were seeded in 24-well plate and cultured for 24 h, and then 200 μl pipet tip was used to scratch the cell layers. The medium was then substituted with fresh culture medium, culture medium with 300 μM H_2_O_2_, culture medium with 300 μM H_2_O_2_ and 50 μl CSOD-BFZ hydrogel and culture medium with 300 μM H_2_O_2_ and 50 μL CFOD-BFZ hydrogel in each group. The cells were continuously cultured and photographed at the specific time points. The ratio of migration was statistically analysed by Image J.

### Assays of the protective effect of CFOD-BFZ hydrogel on H9C2 cells under oxidative microenvironment

The protective effect of CFOD-BFZ hydrogel on H9C2 cells under oxidative stress microenvironment simulated by H_2_O_2_ was studied *in vitro*. After culturing H9C2 cells in a 96-well plate with the density of 2 × 10^4^ cells for 24 h, the fresh media containing 300 μM H_2_O_2_, 300 μM H_2_O_2_ and 50 μl CSOD-BFZ hydrogel or CFOD-BFZ hydrogel were added to the plate for further culturing for extra 12 h. The cell viability was then determined by using MTT assay as per the method mentioned above.

To detect the intracellular ROS content, H9C2 cells were first cultured in a 24-well plate, followed by treated with 300 μM H_2_O_2_, 300 μM H_2_O_2_ and 50 μl CSOD-BFZ hydrogel or CFOD-BFZ hydrogel, respectively, for another 12 h. And then, the DCFH-DA probes were added to stain the intracellular ROS and the cell nucleus was stained with Hoechst 33342. The images were taken by an inverted fluorescence microscope (Invitrogen, EVOS M5000, USA).

### Observation of the morphology of HUVECs

To judge the occurrence of EndoMT, the phenotype of the HUVECs could be observed through their morphology. The HUVECs were seeded in a 24-well plate at the density of 1 × 10^5^ cells per well, followed by treated with 10 ng/ml TGF-β1 or 10 ng/ml TGF-β1 with 20 μL BFZ NPs or 10 ng/ml TGF-β1 with 50 μl CFOD-BFZ hydrogel. After incubating for 72 h, the HUVECs were stained with calcein AM and observed with inverted fluorescence microscope (Invitrogen, EVOS M5000, USA) to judge the phenotypes of the cells.

### Reverse transcription-polymerase chain reaction (RT-PCR) analysis

RT-PCR was conducted to determine the anti-inflammatory effect and EndoMT inhibitory effect of CFOD-BFZ hydrogel. The total cellular RNA was extracted by TRIzol from Raw264.7 or HUVECs and reverse transcribed to cDNA. RT-PCR was performed using SYBR Green QPCR Master Mix on a Light Cycler apparatus (Bio-Rad, CFX-Touch). The sequences of the primers are listed in Supplementary Table S1.

### Quantification of cytokines, enzyme activity and ATP content

The PK enzyme activity, PFK enzyme activity and G6DPH activity in H9C2 cells, the level of TGF-β1 secreted by Raw264.7 macrophages and the intracellular ATP content in H9C2 cells and myocardium tissues were all detected by the corresponding assay kits. All the tests were conducted according to the manufacturers’ instructions.

### Immunofluorescence staining

The immunofluorescent staining was used to characterize the expression of P-selectin, CPT1A, CD31 and CD44 in HUVECs and TGF-β1 in Raw264.7 macrophages, respectively. The cells were washed for three times with PBS and fixed with 4% paraformaldehyde. Then, 0.5% triton ×100 was added to rupture the cell membranes for 10 min, followed by adding 0.5% BSA to block for one hour. The slices were incubated with the primary antibodies overnight, followed by incubation with corresponding secondary antibody for another 4 h at 4 °C, and the nuclei were stained with DAPI. The images were obtained on a laser scanning confocal microscope (Nikon A1R+, Shanghai, China).

### Establishment of acute MI model and *in vivo* therapeutic assay of the CFOD-BFZ hydrogel

All the animal experiments were complied with the guidelines of Tianjin Medical Experimental Animal Care, and animal protocols were approved by the institutional Animal Care and Use Committee of Yi Shengyuan Gene Technology (Tianjin) Co., Ltd (protocol number YSY-DWLL-2024404). The male SD rats were randomly divided into five groups, which were: (1) control group; (2) model group; (3) CSOD hydrogel group; (4) CFOD hydrogel group; (5) CFOD-BFZ hydrogel group. The MI model was performed with the classical ligation operation of the left anterior descending artery (LAD) and the injection dose of the hydrogel was set as 100 μl per sample. After finishing the injection, the rats were observed for 30 min until they could move freely.

The left ventricular functions of the rats were assayed by an echocardiography system (Vevo 2100 Imaging System, Canada) at 0 day, 14 day and 28 day-post-surgery. The typical cardiac functional parameters, which were LV ejection fraction (LVEF), LV fractional shortening (LVFS), LV end-diastolic volume (LVDV) and end-systolic volume (LVSV) were mainly measured.

### *In vivo* histochemical and immunofluorescence analysis

Five days post-surgery, the cardiac samples in each group were collected and sliced into 4 μm thick sections for immunofluorescent staining, including: DHE, CD68, CD206 and Tunel. And the cardiac tissue homogenate was collected to conduct the ATP content assay according to the instruction of the assay kit. Twenty-eight days after surgery, the cardiac tissues were harvested and fixed with 4% paraformaldehyde at 4 °C overnight, embedded in paraffin and sliced into 4 μm thick sections for H&E, Masson’s trichrome and WGA staining and immunofluorescence staining, including α-SMA, vWF, COL-III.

### Statistical analysis

The experiments were analysed by one-way analysis of variance (ANOVA) with Tukey’s *post hoc* test, and data are expressed as means ± standard deviations (SD). Statistical significance was defined as *P < 0.05, **P < 0.01, ***P < 0.001, ****P < 0.0001.

## Results

### Preparation and characterization of BFZ NPs

**Figure 2.**
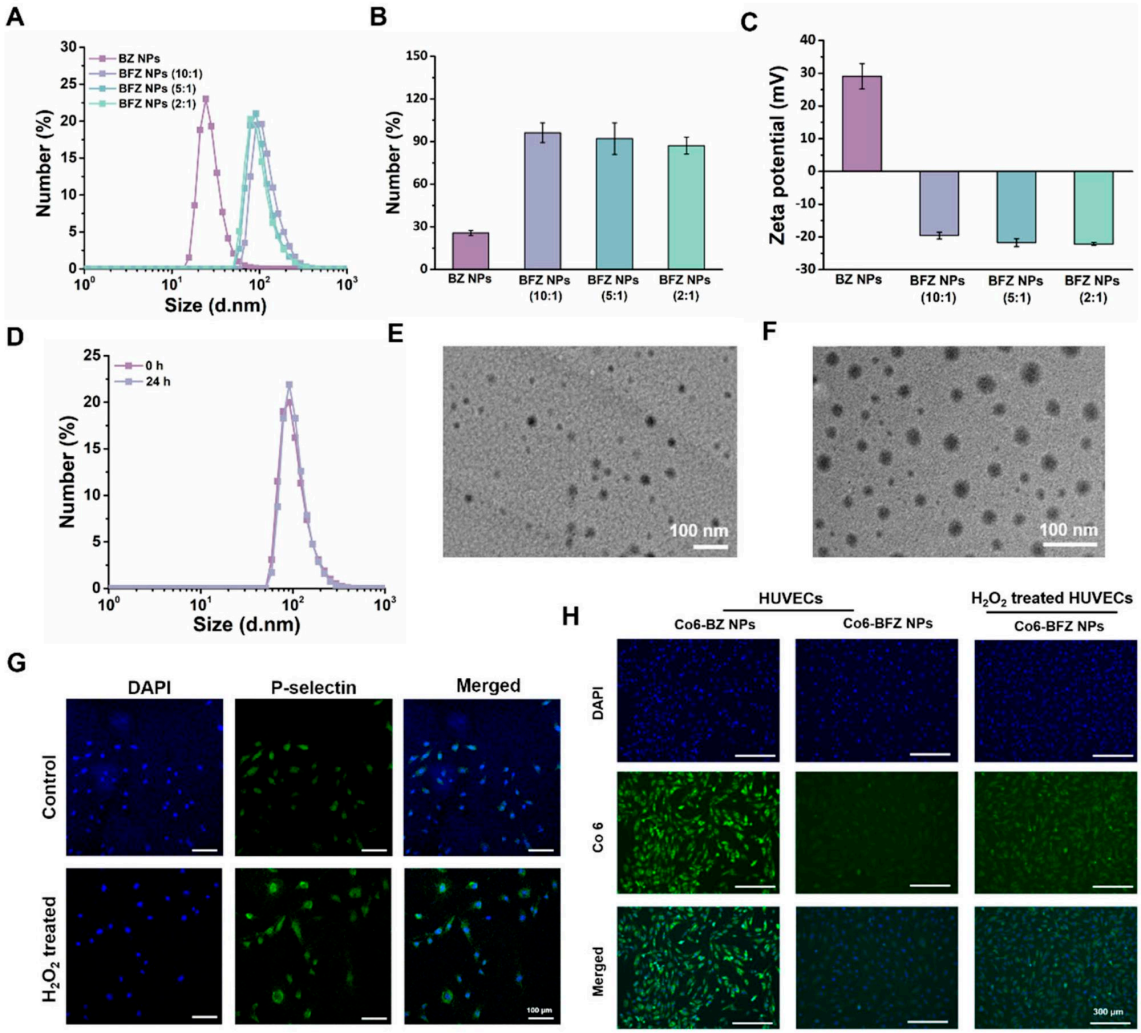
Characterization of the Fu-coated and BA-loaded self-assembled zein nanoparticles (BFZ NPs). (**A–C**) Diameter and zeta potential of the BZ NPs and BFZ NPs measured by DLS. (**D**) Size stability of the BFZ NPs in the mimicked physiological microenvironment. (**E, F**) TEM images of BZ NPs and BFZ NPs. (**G**) Immunofluorescent staining of P-selectin in HUVECs under normal condition and oxidative condition. (**H**) Cellular uptake assays of BZ NPs and BFZ NPs by HUVECs under normal condition and oxidative condition.

Zein, with low solubility in pure water while high solubility in ethanol solution due to the rich amino acid residues in its molecular structure, arises to be an ideal carrier for hydrophobic drugs *via* simple self-assembly. However, the isoelectric point of zein was around 6.2, making the self-assembled nanoparticles unstable in oxidative microenvironment. Therefore, we prepared fucoidan-coated zein nanoparticles for baicalin loading in this study [40]. Zein and BA were first dissolved in 80% (v/v) ethanol solution and then added to water or fucoidan solution dropwise, followed by removing the ethanol by rotary evaporating. Fucoidan could complex on the surface of the zein self-assembled nanoparticles through hydrogen bonds and electrostatic interactions as an anionic polysaccharide, benefiting to improve the stability of the nanoparticles under weak acid microenvironment and provide endothelial-targeting property [9, 37]. We first determined an appropriate mass ratio between zein and fucoidan to obtain nanoparticles with suitable size and zeta potential. As shown in Figure 2A–C, the introduction of fucoidan obviously changed the size and the zeta potential of the original BZ NPs. With the mass ratio of zein to fucoidan varying from 10:1–2:1, the zeta potential of the nanoparticles decreased along with the increase of fucoidan content, and the size of the nanoparticles also decreased slightly due to the increased electrostatic repulsion among different nanoparticles. Finally, the nanoparticles with the mass ratio of 5:1 was fixed because of the moderate particle size and zeta potential (92.03 ± 11.02 nm and 21.76 ± 1.19 mV) for further use (the BFZ NPs in the subsequent experiments all referred to this mass ratio). The BFZ NPs showed stability in simulative microenvironment within 24 h (Figure 2D). The TEM images in Figure 2E and F exhibited the spherical and uniform morphology of the BZ NPs and BFZ NPs, and the size corresponded with the results from DLS tests. The loading capacity and encapsulation efficiency of baicalin in BFZ NPs were calculated as 4.35 ± 0.03% and 44.58 ± 0.33%, respectively.

As for cellular uptake, the BZ NPs with positive zeta potential could be easily internalized by various cells, such as macrophages, H9C2 cells and HUVECs, while the negative zeta potential of BFZ NPs hindered their cellular uptake by macrophages and H9C2 cells (Supplementary Figure S1). However, owing to the P-selectin expressed on HUVECs, which was even upregulated under inflammatory or hypoxia microenvironment, BFZ NPs could specifically bind with HUVECs and then be internalized through the interaction between fucoidan and P-selectin, endowing the nanoparticles with HUVECs-targeted property [9]. The expression of P-selectin on HUVECs under normal or oxidative conditions was verified through immunofluorescent staining (Figure 2G and Supplementary Figure S2), and the cellular uptake images of BZ NPs and BFZ NPs by HUVECs are presented in Figure 2H and the quantitative analysis is shown in Supplementary Figure S3. In normal condition, the expression of P-selectin on HUVECs was not adequate to contend the negative charges of BFZ NPs, resulting in less nanoparticles being internalized by HUVECs. However, the HUVECs expressed more P-selectin under simulative oxidative microenvironment so as to improve the cellular uptake of negative charged BFZ NPs by HUVECs. Thus, we obtained fucoidan-coated zein nanoparticles for baicalin loading *via* simple self-assembly and polysaccharide complexation, and the BFZ NPs possessed stability in mimetic physiological microenvironment and HUVECs-targeted property.

### Preparation and characterization of the injectable nanocomposite hydrogel

In order to obtain an injectable hydrogel with rapid gelation time, bioenergy supplying function and self-immunoregulating effect, the FBP modified carboxymethyl chitosan (CF) and OD were prepared, respectively, as per the methods described in the previous work [3, 30]. The ^1^H NMR spectra, FTIR spectra and XRD results in Supplementary Figure S4 verified the chemical structure of CF. As shown in the ^1^H NMR spectra, the multiple methylene characteristic peaks around 4 ppm primarily confirmed the successful grafting of FBP onto the side chains of CS. As for the FTIR spectra, the decrease of the –NH_2_ characteristic peaks around 3500 and 1590 cm^−1^, the emergency of the C–H characteristic peak around 2900 cm^−1^ and the C=N characteristic peak around 1700 cm^−1^ further verified the successful synthesis of CF [30]. Meanwhile, the crystalline characteristic peak of CS at 20 ° disappeared in CF in the XRD results, further demonstrating the grafting of FBP onto the side chains of CS to disturb its crystal structure. Also, the successful preparation of OD was verified from the peak of C=O at 1730 cm^−1^ in the FTIR spectra in Supplementary Figure S5.

**Figure 3.**
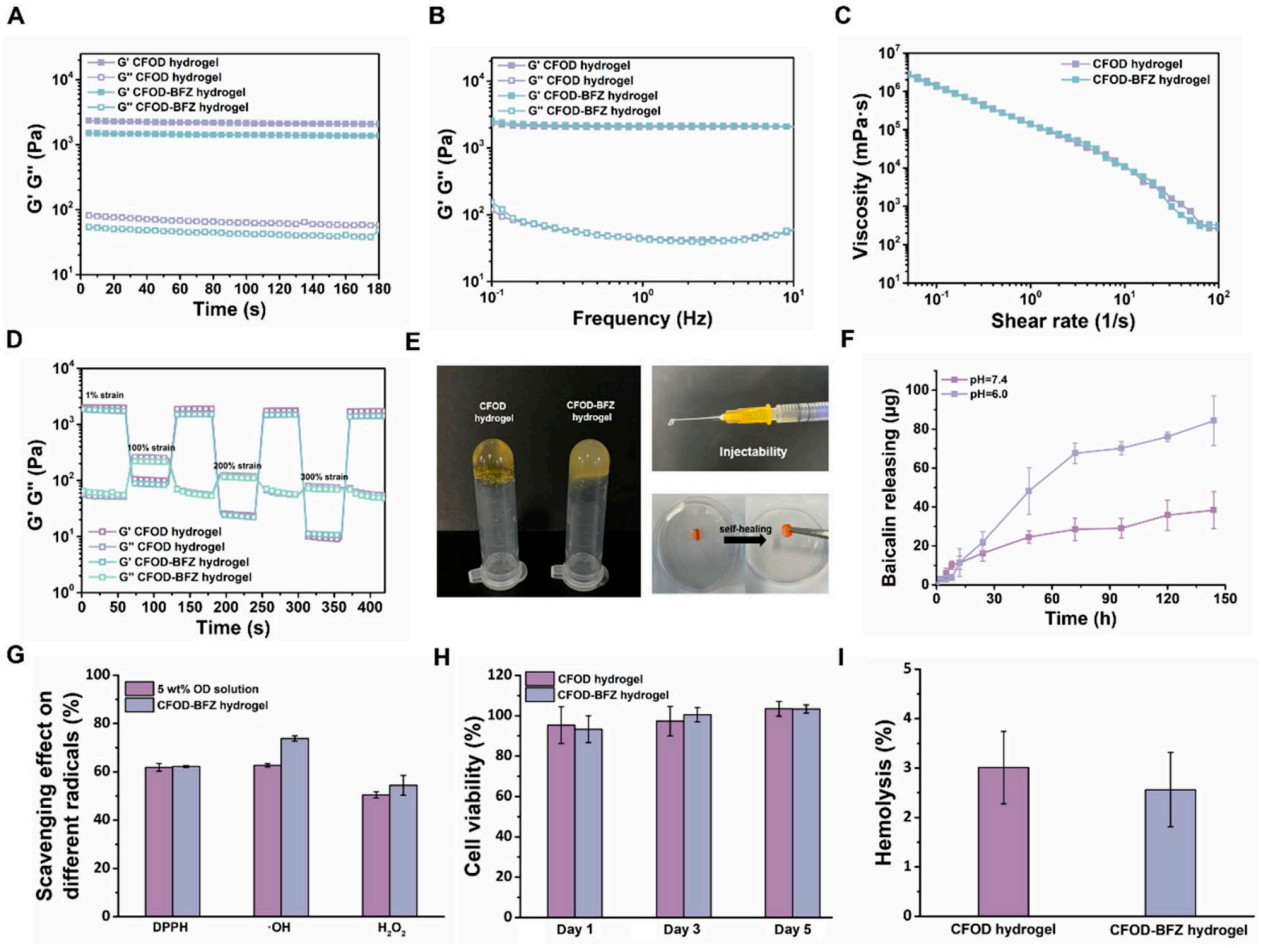
Characterization of the CFOD hydrogel and CFOD-BFZ hydrogel. (**A–D**) Rheological assays of the CFOD hydrogel and CFOD-BFZ hydrogel: time sweeping assay, frequency sweeping assay, shear shinning test and alternate strain sweeping. (**E**) Images of the gelation, injectability and self-healing property of the hydrogels. (**F**) Assays of the BA releasing behavior from CFOD-BFZ hydrogel at pH = 7.4 and pH = 6.0. (**G**) The scavenging effect on different radicals (DPPH, ·OH radicals and H_2_O_2_) of CFOD-BFZ hydrogel. (**H**) Cell viability assays of the CFOD hydrogel and CFOD-BFZ hydrogel at day 1, day 3 and day 5. (**I**) Hemolysis ratio of the CFOD hydrogel and CFOD-BFZ hydrogel.

Subsequently, CF and OD solutions with different concentrations were first mixed to determine an appropriate mass ratio to form a hydrogel with suitable gelation time and storage modulus. The time sweeping results of the hydrogels with different concentrations are presented in Supplementary Figure S6, and the hydrogel formed from 10 wt% CF solution and 5 wt% OD solution (labeled as CFOD hydrogel) was determined for further research, in view of its rapid gelation time and suitable modulus. Next, BFZ NPs were introduced into this system with the concentration of 2.5 mg/ml and the forming hydrogel was named as CFOD-BFZ hydrogel. The gelation time was measured through inverted small bottle method, and the results in Supplementary Figure S7 showed that both of the hydrogels possessed a rapid gelation time within 20 s, which was applicable for *in vivo* injection. And the gelation time of CFOD-BFZ hydrogel slightly increased compared to CFOD hydrogel, which could be attributed to the increased distance among the polymer chains due to the addition of the BFZ NPs. The rheological measurements of CFOD hydrogel and CFOD-BFZ hydrogel were conducted (Figure 3A–D). The time sweeping tests showed that the hydrogels presented modulus of around 1000 Pa, and the frequency tests exhibited that the hydrogels could keep stable in the range of 1–10 Hz to match the rapid beat heart. Both of the hydrogels exhibited shear-thinning property, which was essential for an injectable hydrogel. Furthermore, the hydrogels could rapidly respond to the change of strain and convert into gel state when the applied strain decreased from 100–300% to 1%, which confirmed the self-healing capacity of the hydrogels. Some extra images in Figure 3E further verified the gelation, injectability and self-healing capacity of the hydrogels.

The drug releasing behaviors of BA are investigated in Figure 3F. The condition of pH = 7.4 mimicked the normal physiological environment and the condition of pH = 6.0 mimicked the *in vivo* weak acid inflammatory microenvironment. BA showed a sustained releasing behavior from both of the hydrogels, which meet the need to load the hydrophobic drug with nanoparticles. Since the hydrogel was crosslinked *via* Schiff base reaction between amino groups and aldehyde groups, which was unstable and ease to decompose in weak acid condition [41], BA released more rapidly in the mimicked inflammatory microenvironment, benefiting its sustainable release when being applied into myocardium. Moreover, dextran with 40 kDa molecular weight has been studied to show a satisfying antioxidant property to several radicals due to its rich hydroxyl groups in its structure [28], making dextran arise to be an ideal material to construct self-immunoregulating hydrogel. The antioxidant properties of the hydrogel were further verified, and the OD solution was set as control since the antioxidant ability was mainly provided by OD. Corresponding with the ROS scavenging effect presented by OD solution alone, the results in Figure 3G showed that the CFOD-BFZ hydrogel possessed satisfying scavenging effects to DPPH radicals, ·OH radicals and H_2_O_2_ radicals, confirming the self-immunoregulating effect of the hydrogel. Additionally, the biocompatibility of the hydrogels was tested and the results are presented in Figure 3H and I and Supplementary Figure S8. The cytotoxicity tests verified that the hydrogels showed no adverse effect to cellular proliferation in the period of 5 days, and the hemolysis tests further verified the biosafety of the hydrogels. The *in vivo* degradation behavior of the CFOD-BFZ hydrogel was also investigated *via* subcutaneous implantation. The results in Supplementary Figure S9 showed that the hydrogel presented gradual degradation behavior within 14 days and it nearly totally degraded at day 14, demonstrating better biodegradability for *in vivo* application. In summary, an injectable nanocomposite hydrogel with biocompatibility was formed in a mild and rapid way, by which *in situ* injection of FBP could be achieved through the component of CF in the hydrogel, and antioxidant and anti-inflammatory effect could be directly included *via* the intrinsic property of OD, and the HUVECs-targeted delivery of therapeutic drug was also integrated in this hydrogel system through the introduction of BFZ NPs.

### The CFOD-BFZ hydrogel regulating glycolysis and enhancing viability of cardiomyocytes

**Figure 4.**
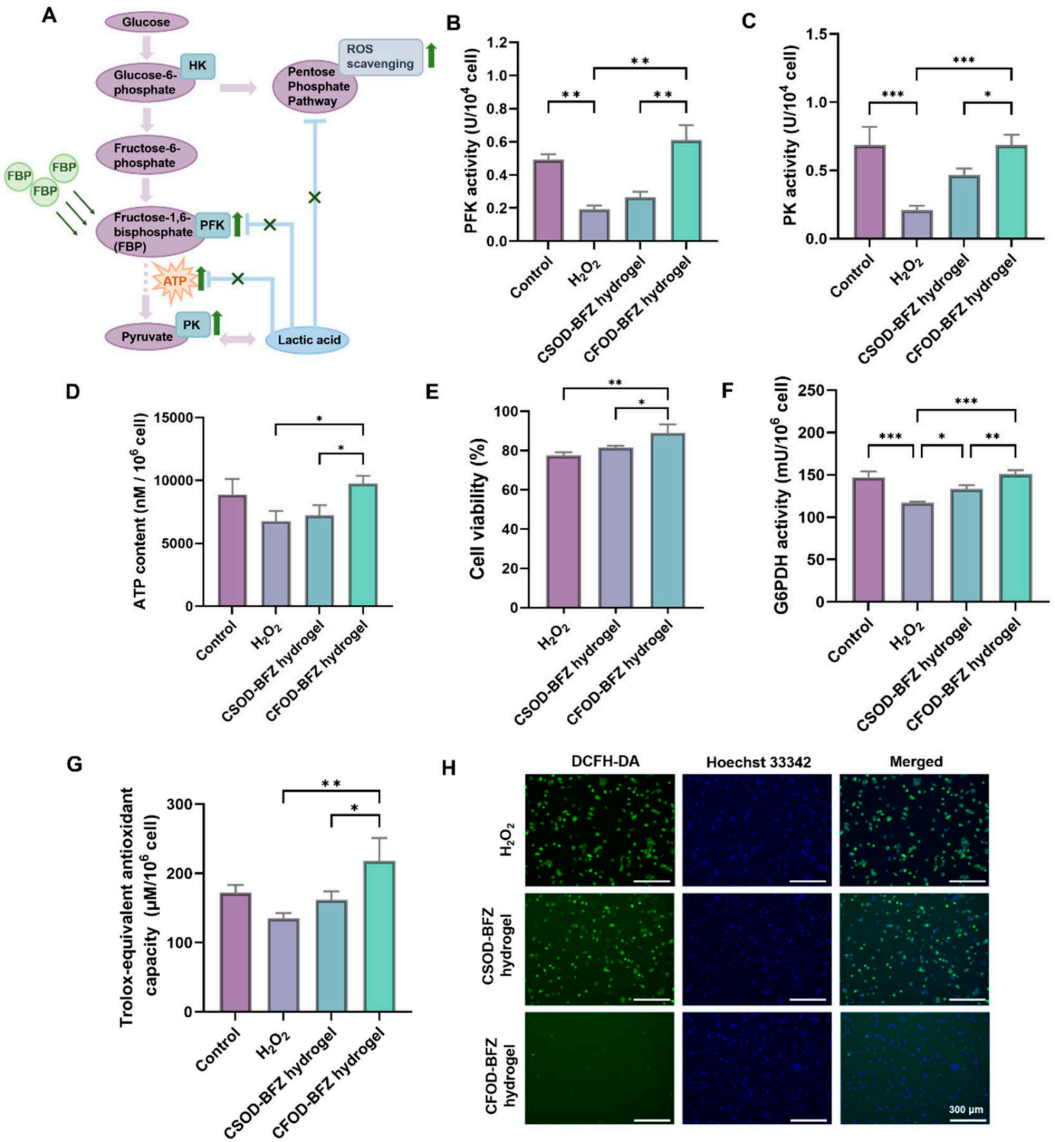
Characterization of the glucose metabolism regulating effect on cardiomyocytes of CFOD-BFZ hydrogel. (**A**) Schematic illustration of the mechanism for FBP release from CFOD-BFZ hydrogel to regulate the glucose metabolism and enhance ATP production. (**B–D**) Assays of the PK, PFK activity and the content of ATP in cardiomyocytes. (**E**) Cell viability assay of the cardiomyocytes treated with CSOD-BFZ hydrogel or CFOD-BFZ hydrogel under oxidative stress. (**F**) Assay of the activity of G6PDH activity in cardiomyocytes treated with CSOD-BFZ hydrogel or CFOD-BFZ hydrogel under oxidative stress. (**G**) Total antioxidant capacity of cardiomyocytes treated with CSOD-BFZ hydrogel or CFOD-BFZ hydrogel under oxidative stress. (**H**) Intracellular ROS level of cardiomyocytes treated with CSOD-BFZ hydrogel or CFOD-BFZ hydrogel under oxidative stress detected by DCFH-DA probe (*P < 0.05, **P < 0.01, ***P < 0.001, *n* = 3).

Once experiencing MI, the cardiomyocytes would face severe ischemia and hypoxic microenvironment, hindering their aerobic glucose metabolism and making them rely more on glycolysis pathway. However, the glycolysis alone would produce less ATP for cellular energy supplying, which ultimately lead to the apoptosis of cardiac myocytes post-MI. Moreover, the intracellularly accumulated lactic acid as the production of glycolysis would decrease the activity of PFK and PK, disturb the pentose phosphate pathway to scavenge intracellular free radicals, further affecting the production of ATP and accelerating cellular apoptosis. When exogenously supplementing FBP from CFOD-BFZ hydrogel into cardiomyocytes, serving as a high-energy intermediate in glucose metabolism, it would directly regulate the glycolysis back to the normal level to restore ATP supplying by enhancing the activity of PFK and PK, and improve the pentose phosphate pathway at the same time to better eliminate the excessive intracellular ROS, thus rescuing the myocardium from oxidative stress comprehensively. The underlying mechanism of exogenous addition of FBP from CFOD-BFZ hydrogel to regulate glucose glycolysis of cardiomyocytes is present in Figure 4A. The specific functions were then verified. H9C2 cells were treated with H_2_O_2_ to mimic the oxidative microenvironment *in vitro* post-MI. The CSOD-BFZ hydrogel (consisting of CS and OD, and lacking of FBP) was specially set for control. (The rheological characterizations of CDOD-BFZ hydrogel are present in Supplementary Figure S10 to exclude other influence factor as control group.) The results in Figure 4B–D verified that the CFOD-BFZ hydrogel significantly improved the level of PFK and PK (the key enzymes for glycolysis) by providing FBP intracellularly, thus increasing the production of ATP for cellular energy supplying, while the CSOD-BFZ hydrogel showed no obvious effects on improving cellular glycolysis due to the lack of providing FBP to the cardiomyocytes. Once the cellular glycolysis was regulated to the normal level, the cardiomyocytes could be saved from apoptosis with abundant energy supplying. As per the result shown in Figure 4E, compared with the cells in the control groups, the cells treated with CFOD-BFZ hydrogel showed a higher cellular viability (more than 80%) under oxidative stress. Additionally, the results of the scratch wound healing test in Supplementary Figure S11 also presented the raised proliferating capacity of H9C2 cells after treated with CFOD-BFZ hydrogel under oxidative microenvironment. Meanwhile, the provided FBP also restored the level of pentose phosphate pathway, which was verified by the improved G6PDH activity, a key enzyme of pentose phosphate pathway, in Figure 4F. A normal pentose phosphate level subsequently recovered the intracellular oxidation-reduction (REDOX) homeostasis, facilitating the scavenging of the excessive intracellular ROS. The results in Figure 4G exhibited that the CFOD-BFZ hydrogel showed benefits in elevating the cellular total antioxidant capacity. And using DCFH-DA as a ROS probe, the intracellular ROS level significantly decreased after treated with CFOD-BFZ hydrogel under oxidative microenvironment, verifying a balanced REDOX after regulating the glucose metabolism of the cardiac myocytes (Figure 4H and Supplementary Figure S12). In conclusion, compared with some other complicated methods, the CFOD-BFZ hydrogel achieved satisfying bioenergy supplying effect in a more convenient way, which could provide high-energy intermediate of glucose metabolism—FBP to cardiomyocytes, regulating glycolysis to enhance the ATP supplying for cellular viability and restoring the pentose phosphate pathway to improve intracellular REDOX homeostasis.

### The CFOD-BFZ hydrogel regulating inflammation and attenuating EndoMT

**Figure 5.**
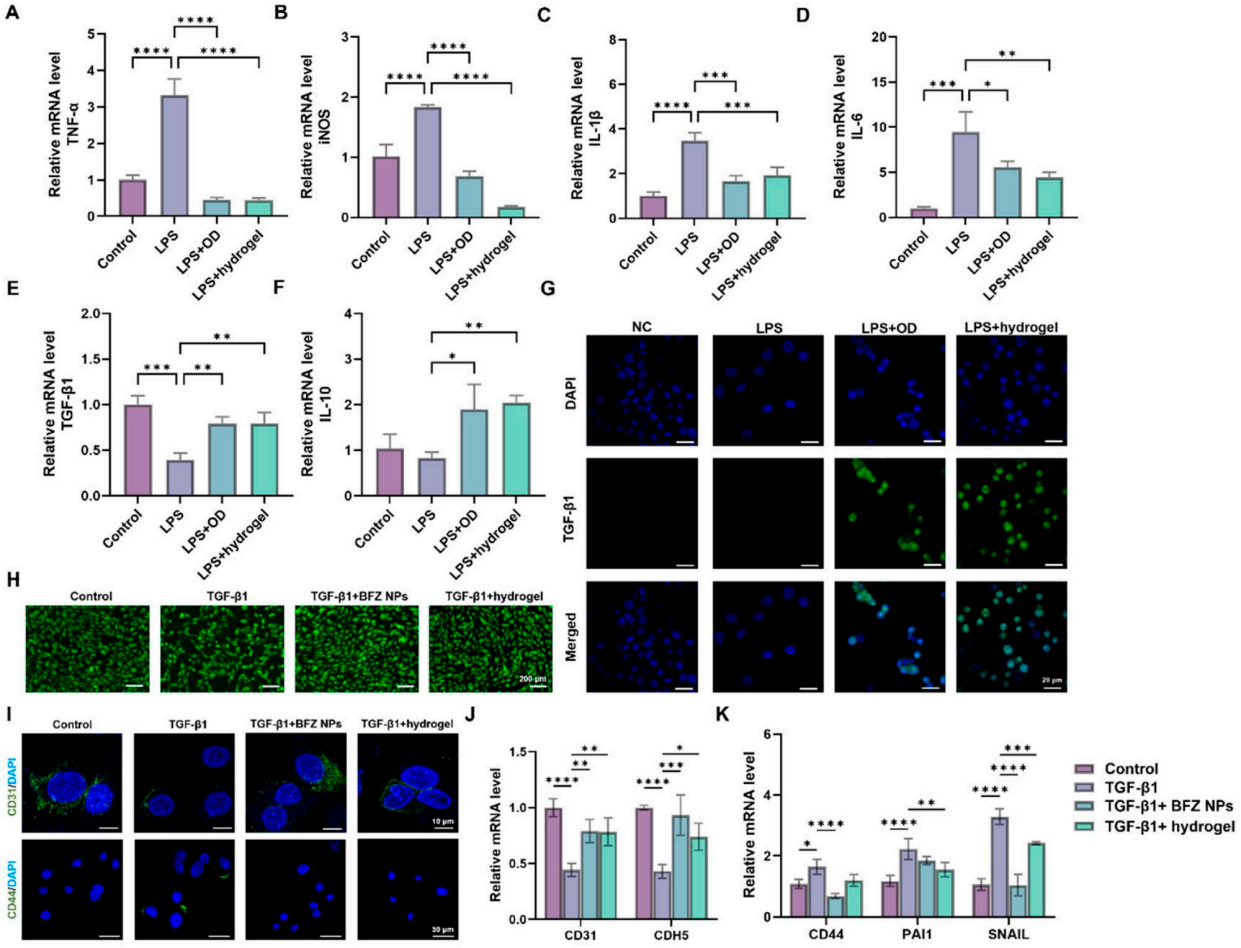
Characterization of the anti-inflammatory effect and attenuating EndoMT function of CFOD-BFZ hydrogel. (**A–D**) Relative mRNA expression of pro-inflammatory genes (TNF-α, iNOS, IL-6 and IL-1β) in macrophages treated with LPS, OD solution and CFOD-BFZ hydrogel. (**E, F**) Relative mRNA expression of anti-inflammatory genes (IL-10 and TGF-β1) in macrophages treated with LPS, OD solution and CFOD-BFZ hydrogel. (**G**) Immunofluorescent staining of TGF-β1 in macrophages treated with OD solution and CFOD-BFZ hydrogel. (**H**) Morphological observation of the HUVECs treated with TGF-β1, BFZ NPs and CFOD-BFZ hydrogel. (**I**) Immunofluorescent staining of CD31 and CD44 in HUVECs treated with TGF-β1, BFZ NPs and CFOD-BFZ hydrogel. (**J, K**) Relative mRNA expression of endothelial genes (CD31 and CDH5) and mesenchymal genes (CD44, PAI1 and SNAIL) in HUVECs treated with TGF-β1, BFZ NPs and CFOD-BFZ hydrogel (*P < 0.05, **P < 0.01, ***P < 0.001, ****P < 0.0001, *n* = 3).

According to the pathological process post-MI, inducing the transition of macrophages from M1 phenotype to M2 phenotype to secrete anti-inflammatory cytokines was essential for initiating proliferative phase of the myocardium. However, the excessive secreted pro-inflammatory cytokines from M1 macrophages usually led to uncontrolled and prolonged inflammation phase, disturbing the regular cardiac reparative period. Thus, anti-inflammatory measures were indispensable in repairing injured myocardium. Compared with the small molecular anti-inflammatory drugs, utilizing self-immunoregulating hydrogels has arisen to be potent strategies due to their sustainable and effective *in situ* administration. In this study, the OD in CFOD-BFZ hydrogel was endowed with the function of immune-regulation for its intrinsic anti-inflammatory function that was investigated *via* blocking NF-κB and inhibiting the phosphorylation of JNK MAPK signal pathways [29]. This could avoid using small molecular anti-inflammatory drugs with short half-life period, and its effects were verified in the following experiments. In Figure 5A–F, the RT-PCR results fully confirmed that OD possessed the capacity of inflammatory regulation, with inhibited expression of pro-inflammatory genes (TNF-α, iNOS, IL-1β and IL-6) and elevated expression of anti-inflammatory genes (IL-10 and TGF-β1), which meant the phenotype of macrophages had been successfully converted into M2 phenotype. Likewise, the CFOD-BFZ hydrogel showed satisfying effects on inducing macrophages toward M2 phenotype to exhibit its anti-inflammatory property under the synergetic functions of OD and CF. The immunofluorescent staining results in Figure 5G further verified that the CFOD-BFZ hydrogel improved the expression of TGF-β1 protein in macrophages, which corresponded with the ELISA test result in Supplementary Figure S13. Thus, the CFOD-BFZ hydrogel showed advantages in regulating the inflammatory phase to a normal period and facilitating the initiation of the subsequent proliferative phase for cardiac regeneration.

However, the abundant expressed TGF-β1 had been reported to lead to the occurrence of EndoMT, which meant that the endothelial cells lost their original morphological characteristics and altered into mesenchymal cells, bringing about the risks of cardiac fibrosis in cardiac remodeling process. According to the involved pathways and underlying mechanisms of TGF-β1-induced EndoMT being reported in the previous work [15], delivering BA into endothelial cells to restore CPT1A activity and modulating FAO metabolism process was expected to be an effective measure for interrupting the unexpected EndoMT. Thus, we verified the treating effects of the prepared BFZ NPs and CFOD-BFZ hydrogel since they have been confirmed to have the functions of targeting HUVECs and intracellularly delivering BA in the experiments above. The immunofluorescent staining results of CPT1A protein in HUVECs are exhibited in Supplementary Figure S14. Corresponding with the expected function of BA, the BFZ NPs and CFOD-BFZ hydrogel elevated the activity of CPT1A under the treatment of TGF-β1, successfully driving the FAO process. As shown in Figure 5H, the endothelial cells treated with TGF-β1 gradually transformed their morphologies and showed mesenchymal features to some extent. However, after treating the endothelial cells with BFZ NPs and CFOD-BFZ hydrogel, the released BA could help resist the alternation of cellular phenotypes and remain their morphological characteristics of endothelial cells. The same conclusions could be drawn from the immunofluorescent staining of CD31 or CD44 in Figure 5I, which were the specific markers of endothelial cells and mesenchymal cells, respectively. The cells treated with TGF-β1 showed stronger immunofluorescent intensity in CD44 staining, while the cells treated with BFZ NPs and CFOD-BFZ hydrogel presented more similar characters with the control group, verifying the inhibition of the EndoMT process. Additionally, we investigated expression of some endothelial related genes and mesenchymal related genes. The results in Figure 5J showed that TGF-β1 obviously elevated the expression of mesenchymal related genes (CD44, PAI1 and SNAIL) and decreased the expression of endothelial related genes (CD31 and CDH5), signifying the occurrence of EndoMT. While the BA released from BFZ NPs and CFOD-BFZ hydrogel successfully attenuated the progress of EndoMT with improved expression of endothelial related genes and depressed expression of mesenchymal related genes in HUVECs. Thus, the CFOD-BFZ hydrogel showed advantages in inhibiting the unexpected EndoMT induced from the excessive secreted anti-inflammatory cytokines by delivery of BA, and reducing the occurrence of cardiac fibrosis in cardiac reparative phase. Among various pathogenic factors of cardiac fibrosis, the design of CFOD-BFZ hydrogel presented a novel treating strategy to attenuate cardiac fibrosis and also break the unexpected relevance between inflammatory reactions and adverse cardiac remodeling.

### *In vivo* therapeutic effects of CFOD-BFZ hydrogel on MI

**Figure 6.**
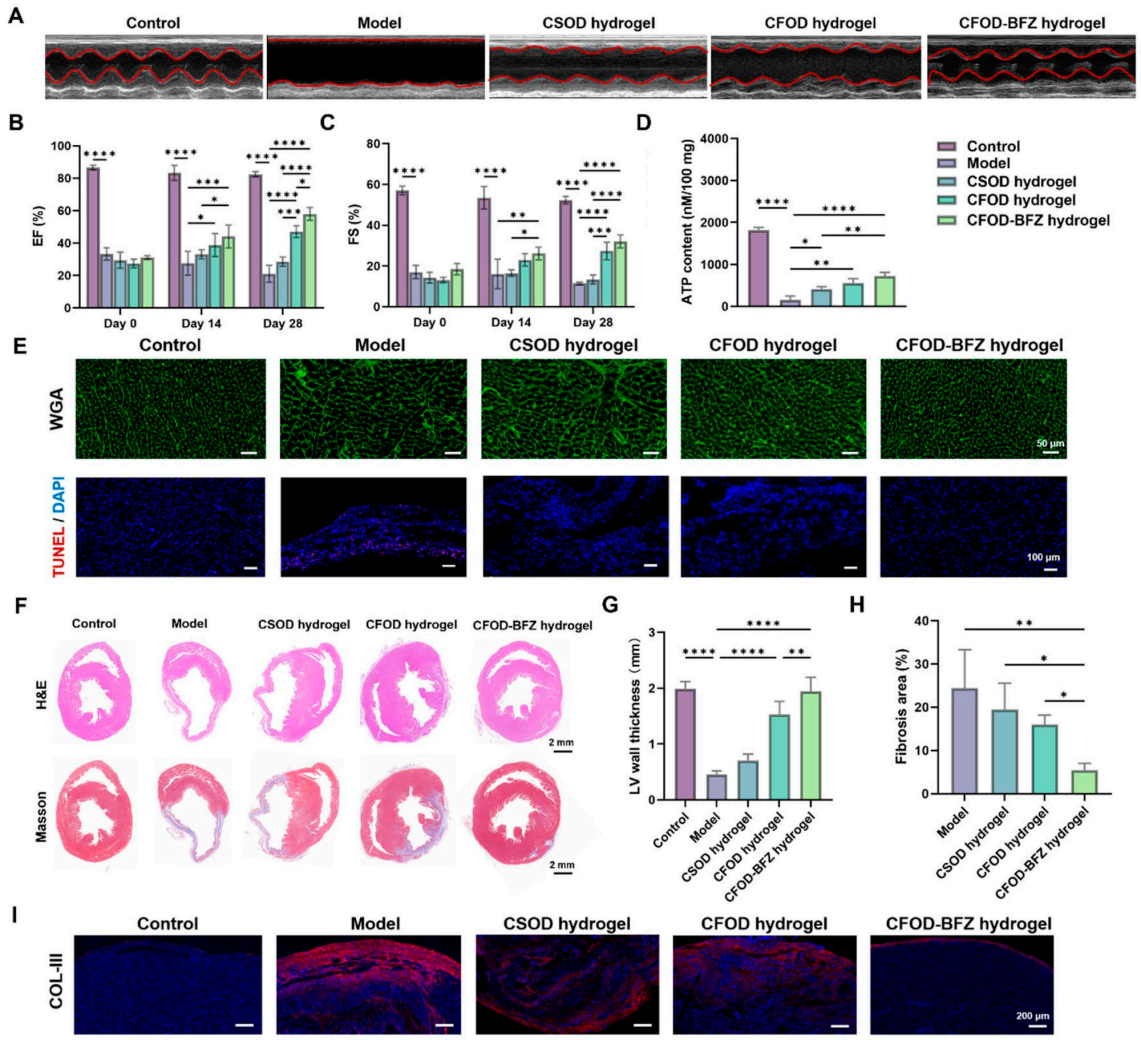
*In vivo* therapeutic effect of CFOD-BFZ hydrogel post-MI. (**A**) Representative images of echocardiography in different groups at day 28-post-surgery. (**B, C**) Statistical analysis of cardiac functions in different groups at 0, 14 and 28 days after surgery. (**D**) Assays of ATP content in the cardiac tissues of different groups. (**E**) Immunofluorescent staining of WGA and Tunel of the cardiac tissues in different groups. (**F**) H&E and Masson staining of the cardiac tissues in different groups at 28 days post-MI. (**G, H**) Statistical analysis of LV wall thickness and fibrosis area of different groups at 28 days after surgery. (**I**) Immunofluorescent staining of COL-III in different groups at 28 days after surgery (*P < 0.05, **P < 0.01, ***P < 0.001, ****P < 0.0001, *n* = 5).

In order to further investigate the *in vivo* therapeutic effects of CFOD-BFZ hydrogel on MI, 50 male rats were randomly and averagely divided into five groups and the MI model was established. All the animal experiments were complied with the guidelines of Tianjin Medical Experimental Animal Care, and animal protocols were approved by the institutional Animal Care and Use Committee of Yi Shengyuan Gene Technology (Tianjin) Co., Ltd (protocol number YSY-DWLL-2024404). The rats were, respectively, administrated by CSOD hydrogel (consisted of CS and OD), CFOD hydrogel and CFOD-BFZ hydrogel, and the control group and the model group (untreated) were set for comparison. Echocardiography was conducted on each group at day 0, day 14 and day 28 post-surgery, and the images and statistical analysis results are presented in Figure 6A–C. The echocardiographic images in Figure 6A primarily verified that the cardiac functions of the rats treated with CFOD-BFZ hydrogel had been restored to a great extent at day 28 post-MI. The statistical analysis results in Figure 6B and C further presented the detailed variation trend of left ventricular ejection fraction (EF) and fractional shortening (FS) during cardiac repairing. In the model group, the cardiac functions of MI rats without any therapy became worse and worse. Although the CSOD hydrogel increased EF and FS values to some extent, the therapeutic effect was not obvious due to the single function of CSOD hydrogel. On the contrary, CFOD hydrogel and CFOD-BFZ hydrogel gradually improved the cardiac functions during cardiac repairing owing to the synergistic effects of FBP to rescue the cardiomyocytes and OD to regulate inflammatory response. Ultimately the cardiac functions after 28 days were remarkably elevated.

The ATP contents of the infarcted myocardial tissues in each group at 5 days post-surgery are tested in Figure 6D, and the result demonstrated that the FBP released from CFOD hydrogel and CFOD-BFZ hydrogel indeed improved the glycolysis of cardiomyocytes and improved their metabolism for better bioenergy supplying, and thus saved the cardiomyocytes from apoptosis to achieve more effective cardiac restoration. The immunofluorescent staining of WGA and Tunel results shown in Figure 6E and Supplementary Figure S15 further verified the positive effects of CFOD hydrogel and CFOD-BFZ hydrogel on injured cardiomyocytes, that is, the improved glycose metabolism and adequate bioenergy supplying for cardiomyocytes obviously rescued the cells from apoptosis and decreased the risks of myocardial hypertrophy, and further attenuated the occurrence of possible cardiac failure. Subsequently, the H&E staining and Masson staining were used to evaluate the morphology of the cardiac tissues in Figure 6F, and the statistical analysis in Figure 6G and H shows the conditions of left ventricular remodeling in each group. The addition of BFZ NPs in CFOD-BFZ hydrogel to regulate EndoMT in endothelial cells showed advantages in increasing the thickness of left ventricular wall and reducing the cardiac fibrosis area, thus inhibiting the adverse remodeling of left ventricular, which was in agreement with the *in vitro* experimental results. Furthermore, the COL-III immunofluorescent staining results of the cardiac tissues in Figure 6I similarly demonstrated the therapeutic effect of CFOD-BFZ hydrogel to reduce the excessive deposition of extracellular matrix so as to attenuate the occurrence of cardiac fibrosis.

As for regulating tissue microenvironment, the CFOD hydrogel and CFOD-BFZ hydrogel presented comprehensive anti-inflammatory and antioxidant effects under the synergetic functions of OD and FBP (Supplementary Figure S16). The cardiac tissues regulated by CFOD hydrogel and CFOD-BFZ hydrogel exhibited lower ROS level detected by DHE probe, and they could also eliminate inflammation with less M1 macrophages being labeled by CD68 and more M2 macrophages being labeled by CD206. And then the reparative M2 macrophages served to promote angiogenesis by secreting abundant cytokines evaluated by more positive vWF and α-SMA immunofluorescent staining results, which demonstrated the generation of both microvasculature and small arterioles to provide more oxygen and nutrition to the infarcted zones.

## Conclusion

In this study, we designed and prepared an injectable nanocomposite hydrogel with integrated immunoregulatory and *in situ* metabolism regulatory effects, aiming to improve cardiac functions and attenuate cardiac fibrosis post-MI. FBP was grafted on the side chains of carboxymethyl chitosan for achieving *in situ* bioenergy supplying, and the 40 kDa dextran with an antioxidant and anti-inflammatory effect was partially oxidized to generate aldehyde groups for crosslinking. Meanwhile, BFZ NPs were loaded for jointly forming the final CFOD-BFZ hydrogel. The FBP released from CF polymer chains had been verified to restore the glycolysis level of cardiomyocytes and enhance the production of ATP for cellular bioenergy supplying *in vitro*. OD exhibited satisfying effects on inducing the polarization of macrophages toward M2 phenotype, promoting the inflammatory phase transit into reparative phase to accelerate cardiac repair. Facing the possibility of EndoMT induced by excessive anti-inflammatory cytokine—TGF-β1, BFZ NPs showed advantages in targeting HUVECs and intracellularly releasing BA for elevating the activity of CPT1A and restoring FAO, thus retaining the endothelial features of HUVECs and attenuating the risk of cardiac fibrosis. After being applied into the infarcted regions of rats, the CFOD-BFZ hydrogel possessed efficient therapeutic effects on raising the viability of cardiomyocytes by enhanced ATP supplying, eliminating excessive inflammatory response and attenuating cardiac fibrosis, further presenting superiorities in reducing the adverse left ventricular remodeling and avoiding unexpected heart failure. This integrated design of self-immunoregulatory and *in situ* metabolism improving injectable hydrogel can be extended to treat several ischemia and inflammatory diseases for facilitating tissue regeneration.

## Supplementary Material

rbae131_Supplementary_Data
