# Supplementary material for: An immunoregulatory and metabolism-improving injectable hydrogel for cardiac repair after myocardial infarction
Source: Regen Biomater. 2024 Nov 13;12:rbae131. doi: 10.1093/rb/rbae131 (PMC11703553; doi:10.1093/rb/rbae131)
Supplement: rbae131_Supplementary_Data [file rbae131_supplementary_data.docx]

**Supporting Information**

**An Immunoregulatory and Metabolism-Improving Injectable Hydrogel for Cardiac Repair After Myocardial Infarction**

Yage Sun, Xinrui Zhao, Qian Zhang, Rong Yang, Wenguang Liu^*^

School of Materials Science and Engineering, Tianjin Key Laboratory of Composite and Functional Materials, Tianjin University, Tianjin 300350, China

Email: [wgliu@tju.edu.cn](mailto:wgliu@tju.edu.cn)


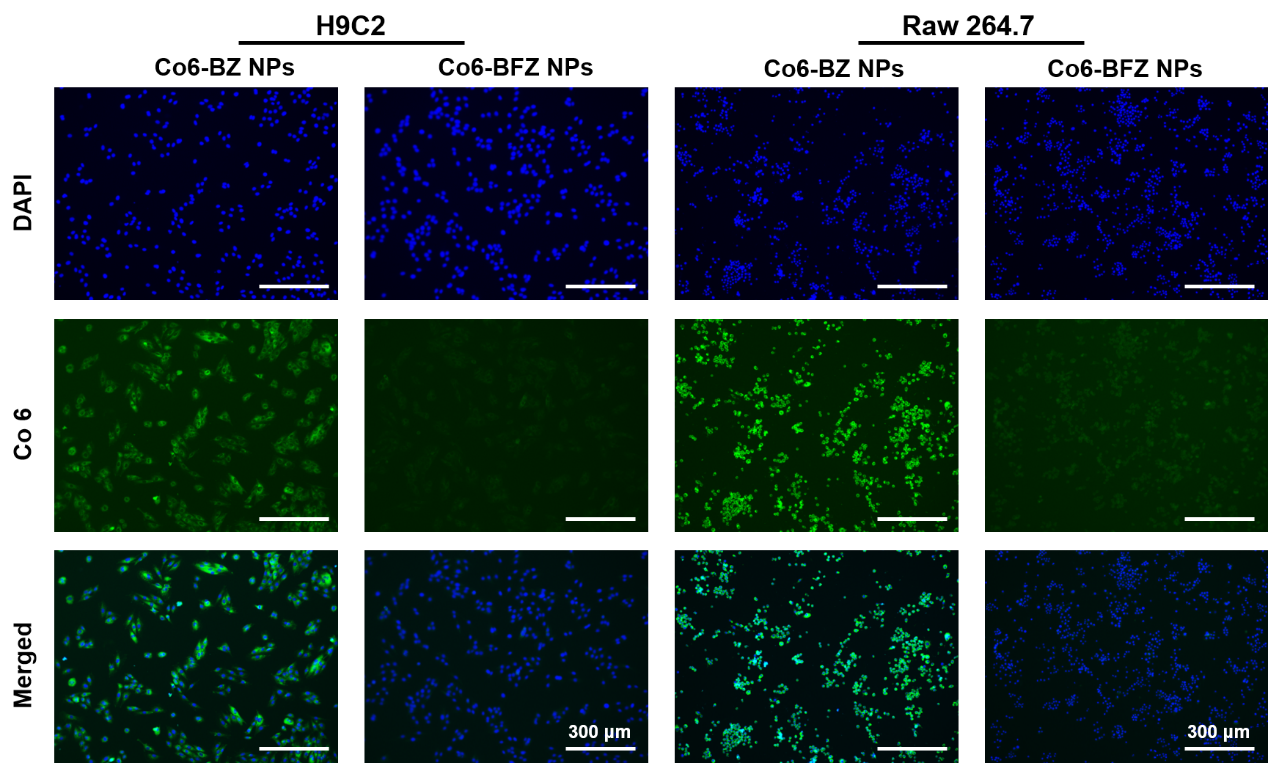


Figure S1. Cellular uptake of BZ NPs and BFZ NPs by H9C2 cells and Raw264.7 macrophages.

Figure S2.Quantitative analysis of immunofluorescence staining of P-selectin. (**P < 0.01, n=3)

Figure S3.Quantitative analysis of the cellular uptake by HUVECs. (**P < 0.01, ****P < 0.0001, n=3)


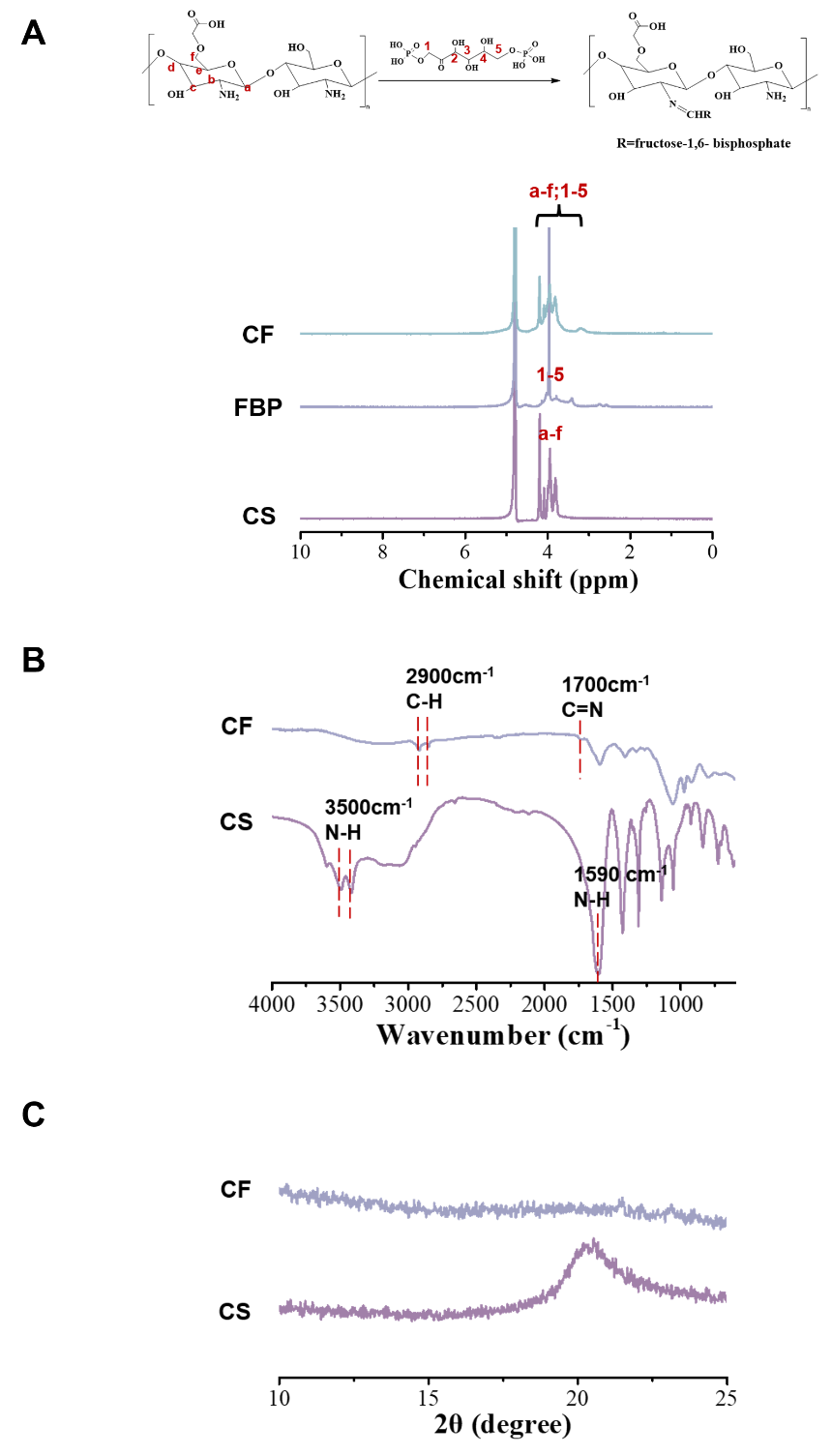


Figure S4. ^1^H NMR spectra (A), FTIR spectra (B) and XRD patterns (C) of CS and CF.


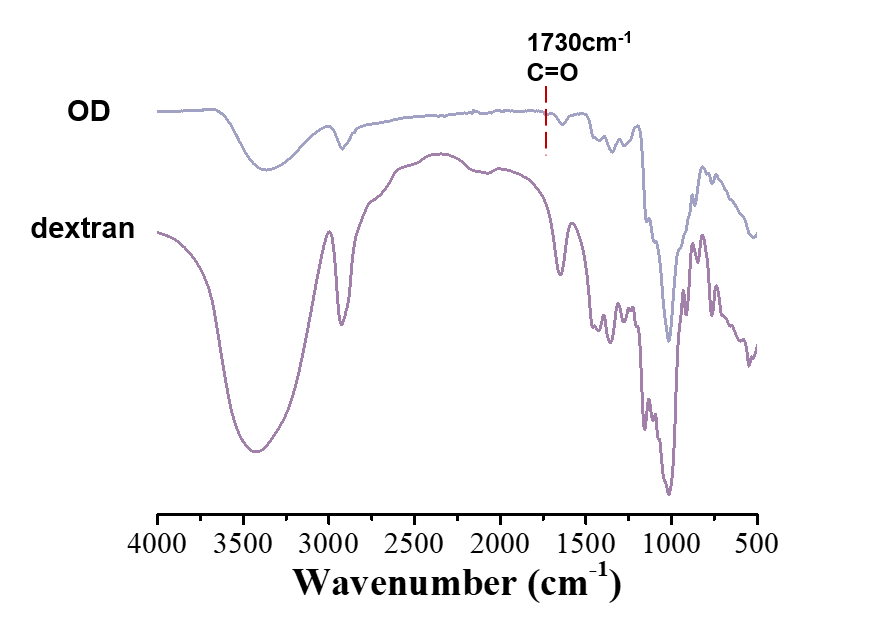


Figure S5. FTIR spectra of dextran and OD.


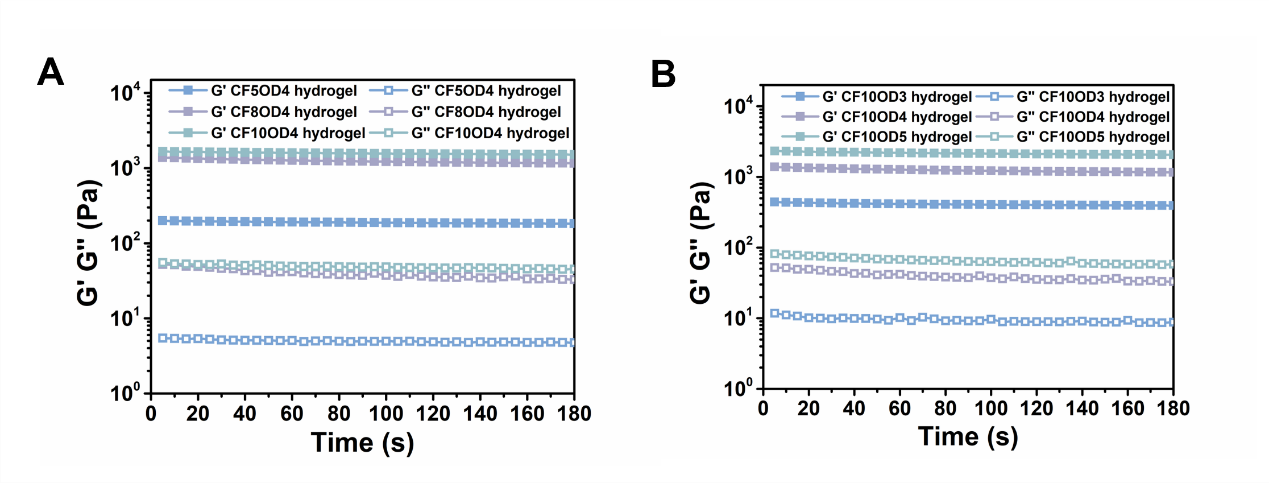


Figure S6. Time sweeping tests of the hydrogels formed from CF solution and OD solution with different concentration.





Figure S7. Gelation time of CFOD hydrogel and CFOD-BFZ hydrogel measured by inverted small bottle method.


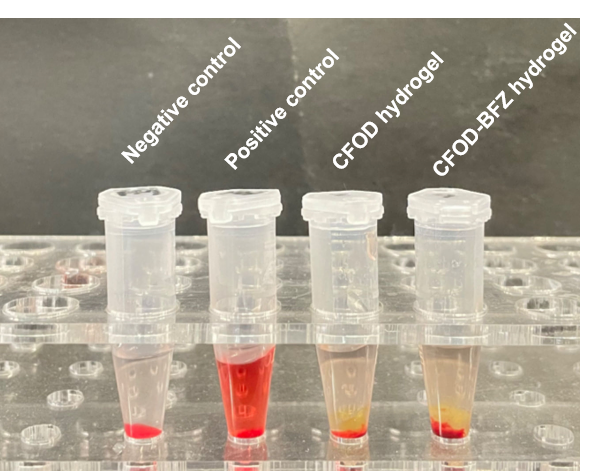


Figure S8. Hemolysis results of CFOD hydrogel and CFOD-BFZ hydrogel.


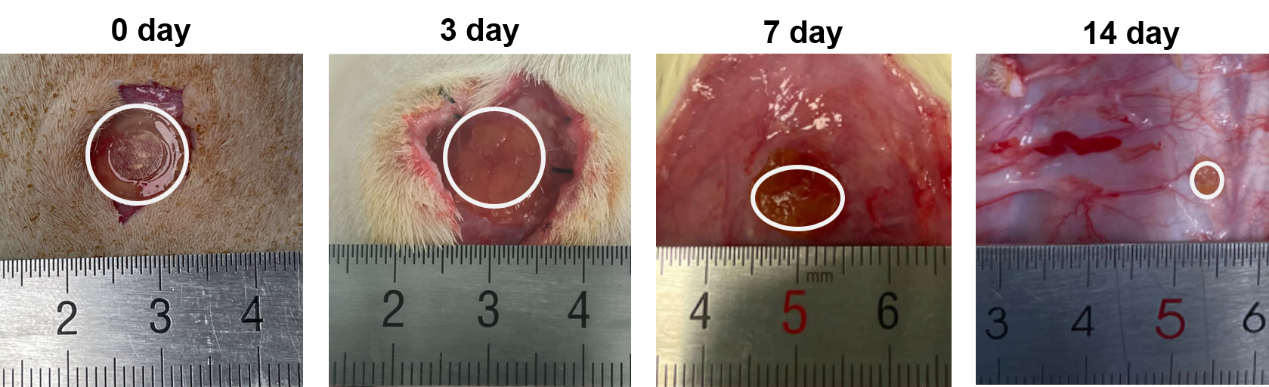


Figure S9. Subcutaneous implantation results of the CFOD-BFZ hydrogel to observe the in vivo degradation behavior.


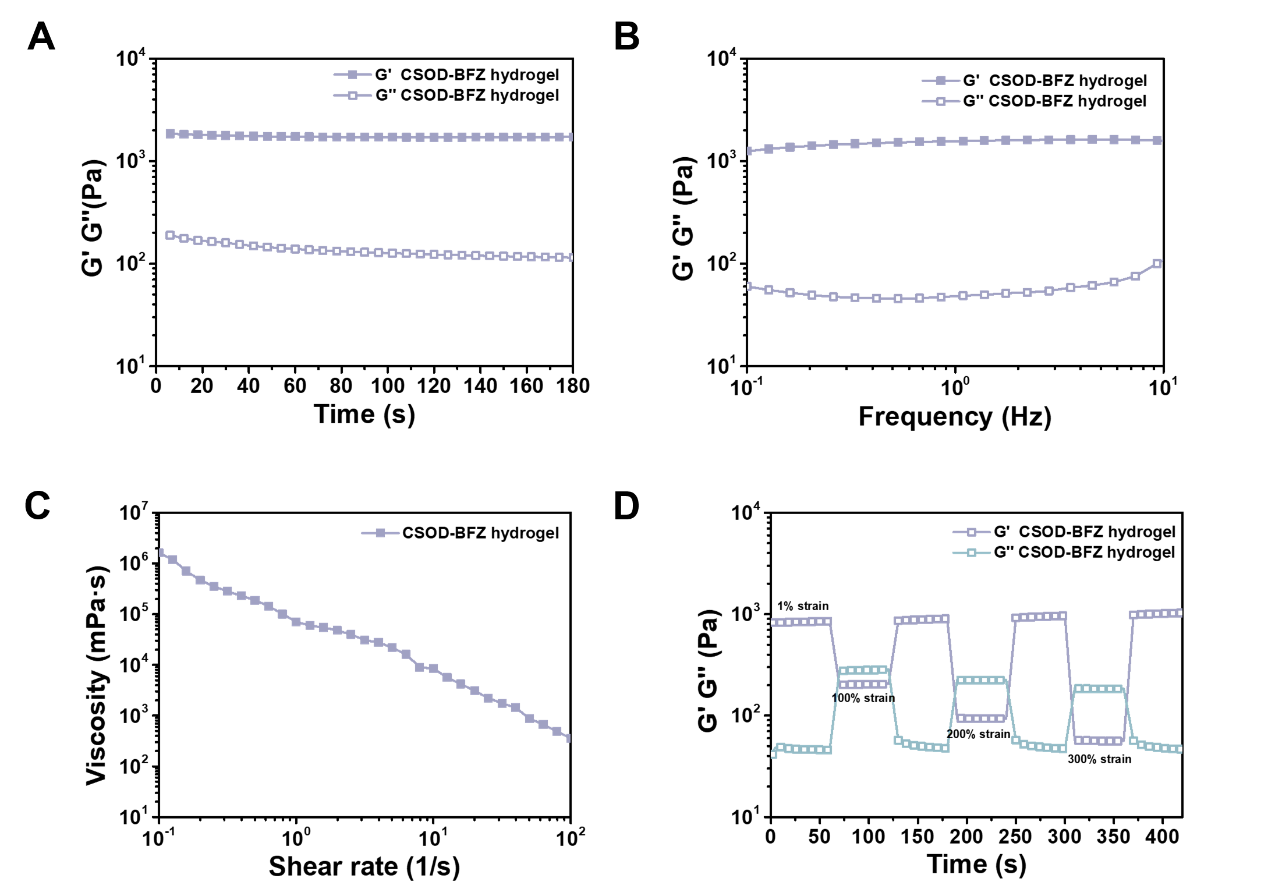


Figure S10. Rheological tests of CSOD-BFZ hydrogel.


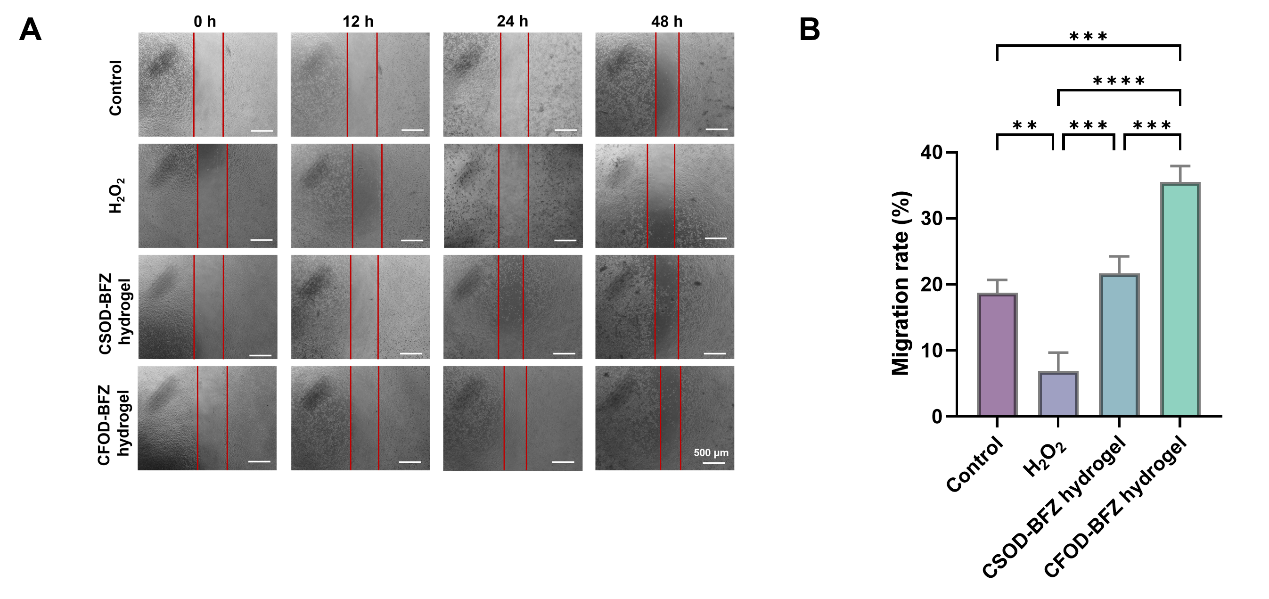


Figure S11. Results of the scratch wound healing tests under oxidative stress and the statistical analysis. (**P < 0.01, ***P < 0.001, ****P < 0.0001, n=3)

Figure S12.Quantitative analysis of the fluorescence staining of DCFH-DA. (*P < 0.05, ****P < 0.0001, n=3)


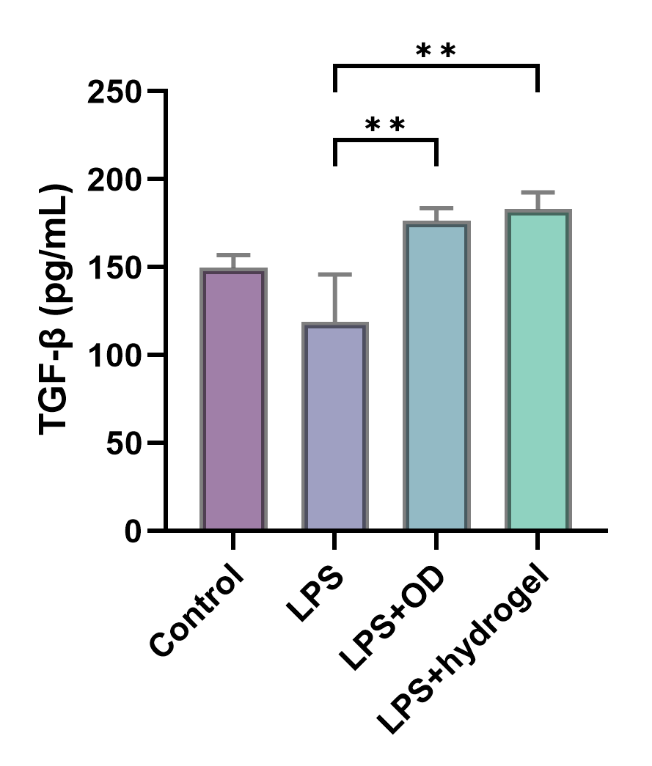


Figure S13. Results of the secretion of TGF-β1 from macrophages by ELISA test. (**P < 0.01, , n=3)


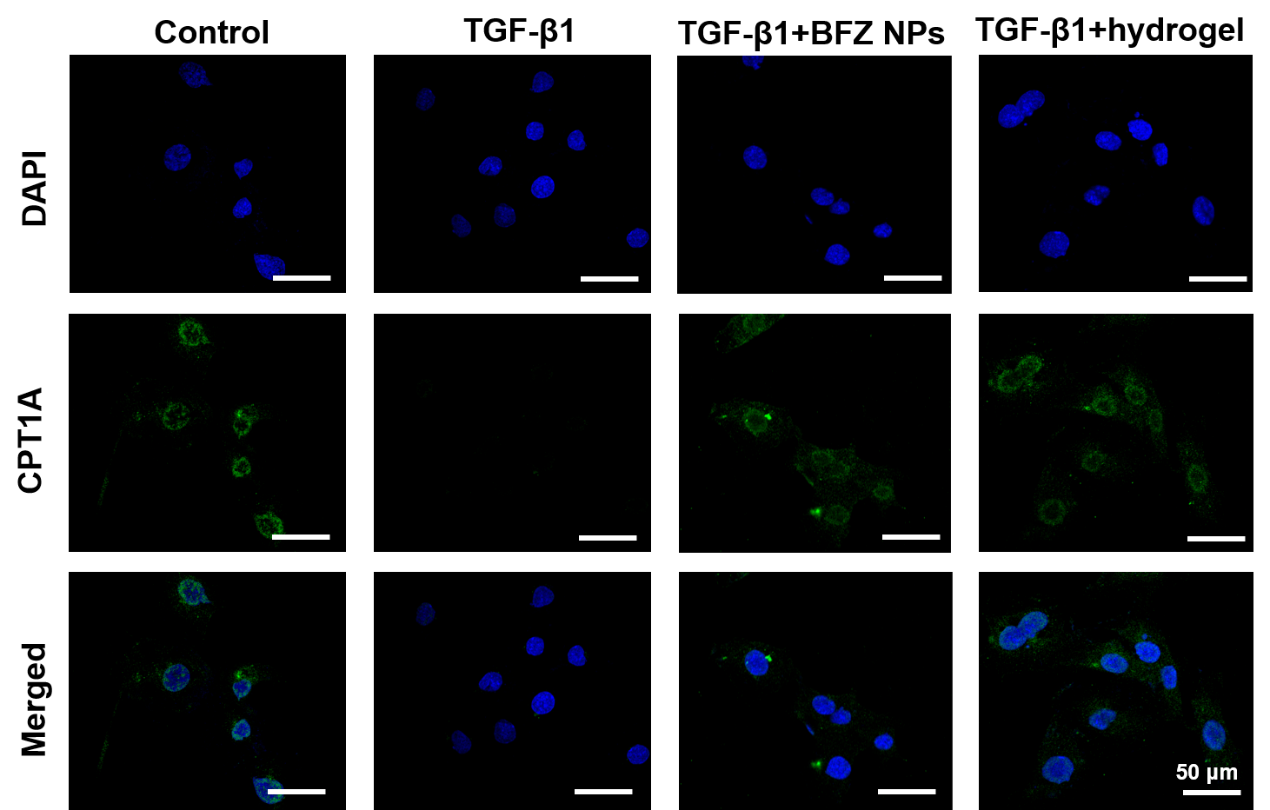


Figure S14. Immunofluorescent staining of CPT1A in HUVECs treated by TGF-β1, BFZ NPs and CFOD-BFZ hydrogel.


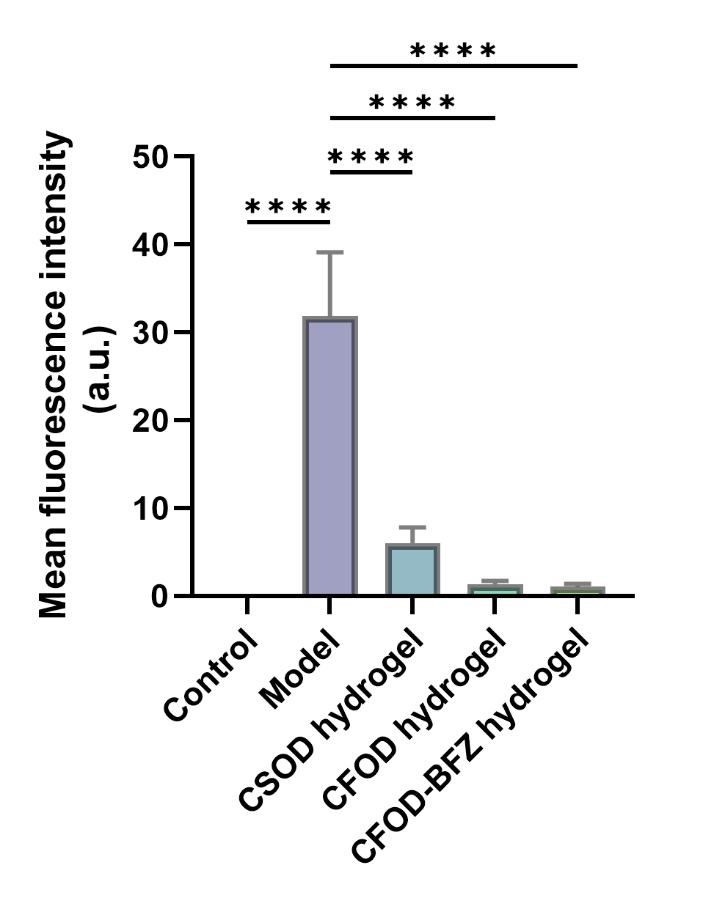


Figure S15. Quantitative analysis of TUNEL immunofluorescent staining. (****P < 0.0001, n=5)


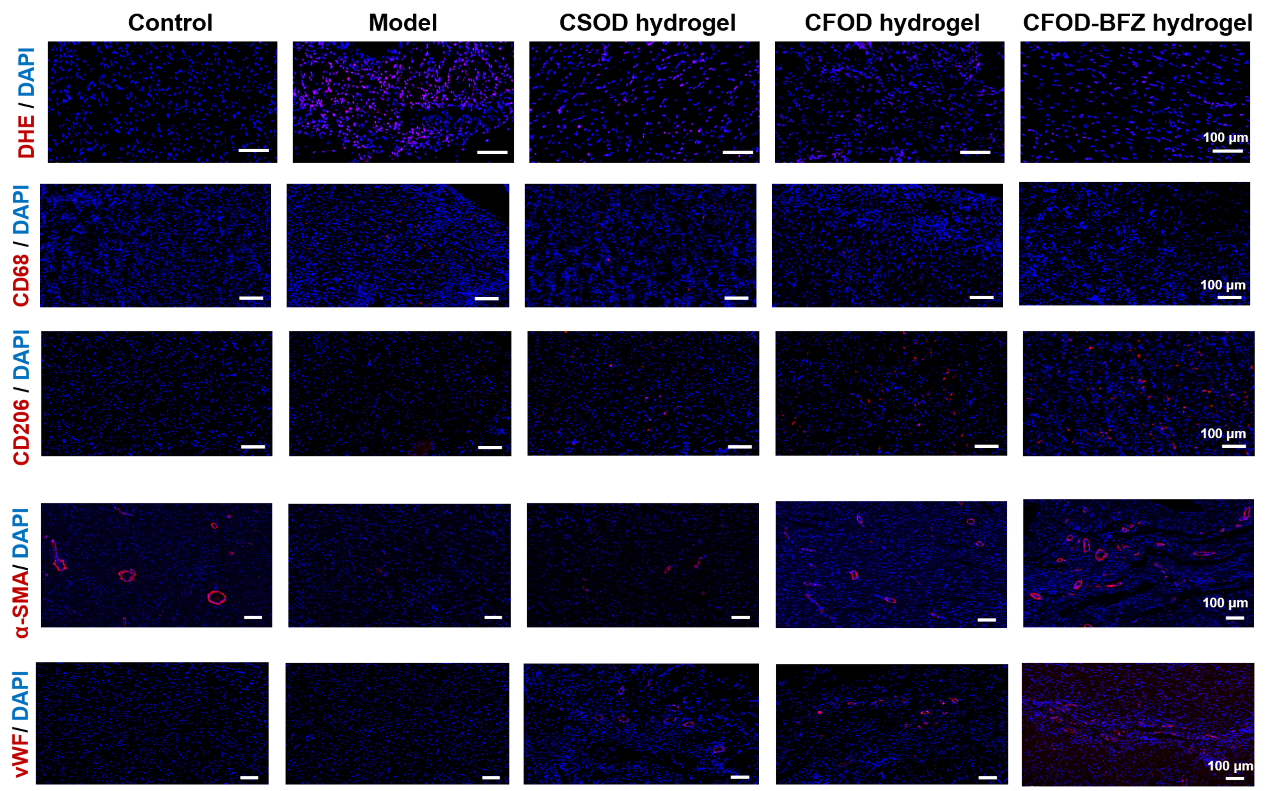


Figure S16. Immunofluorescent staining of DHE, CD68, CD206, α-SMA and vWF of cardiac tissues in different group (n=5).

Table S1 Primer sequences in the RT-PCR studies

| Genes | Sequences |
| --- | --- |
| human GAPDH | F: 5’-AAGGTGAAGGTCGGAGTCAA-3’  R: 5’- AATGAAGGGGTCATTGATGG-3’ |
| human CD44 | F: 5’- GACAAGTTTTGGTGGCACG-3’  R: 5’- CACGTGGAATACACCTGCAA-3’ |
| human PAI1 | F: 5’- ACAACAGGAGGAGAAACCCA-3’  R: 5’- AGCTCCTTGTACAGATGCCG-3’ |
| human SNAIL | F: 5’- CTCTAGGCCCTGGCTGCTAC-3’  R: 5’- TCTGAGTGGGTCTGGAGGTG-3’ |
| human CD31 | F: 5’- CCTTCTGCTCTGTTCAAGCC-3’  R: 5’- GGGTCAGGTTCTTCCCATTT-3’ |
| human CDH5 | F: 5’- AGAGCTCCACTCACGCTCAG-3’  R: 5’- CATCTTCCCAGGAGGAACAG-3’ |
| Mouse GAPDH | F: 5’-AATGCATCCTGCACCACC-3’  R: 5’-ATGCCAGTGAGCTTCCCG-3’ |
| Mouse TNF-α | F: 5’-GACGTGGAACTGGCAGAAGAG-3’  R: 5’-TTGGTGGTTTGTGAGTGTGAG-3’ |
| Mouse iNOS | F: 5’-GTTCTCAGCCCAACAATACAAGA-3’  R: 5’-GTGGACGGGTCGATGTCAC-3’ |
| Mouse IL-6 | F: 5’-TAGTCCTTCCTACCCCAATTTCC-3’  R: 5’-TTGGTCCTTAGCCACTCCTTC-3’ |
| Mouse IL-1β | F: 5’-GCAACTGTTCCTGAACTCAACT-3’  R: 5’-ATCTTTTGGGGTCCGTCAACT-3’ |
| Mouse TGF-β1 | F: 5’- CAGTACAGCAAGGTCCTTGC-3’  R: 5’- ACGTAGTAGACGATGGGCAG-3’ |
| Mouse IL-10 | F: 5’- GCTCTTACTGACTGGCATGAG-3’  R: 5’- CGCAGCTCTAGGAGCATGTG-3’ |
